# Opsonin-Deficient Nucleoproteic Corona Endows UnPEGylated Liposomes with Stealth Properties *In Vivo*

**DOI:** 10.1021/acsnano.1c07687

**Published:** 2022-01-18

**Authors:** Francesca Giulimondi, Elisabetta Vulpis, Luca Digiacomo, Maria Valeria Giuli, Angelica Mancusi, Anna Laura Capriotti, Aldo Laganà, Andrea Cerrato, Riccardo Zenezini Chiozzi, Carmine Nicoletti, Heinz Amenitsch, Francesco Cardarelli, Laura Masuelli, Roberto Bei, Isabella Screpanti, Daniela Pozzi, Alessandra Zingoni, Saula Checquolo, Giulio Caracciolo

**Affiliations:** † Department of Molecular Medicine, Sapienza University of Rome, Viale Regina Elena 291, 00161 Rome, Italy; ‡ Department of Chemistry, Sapienza University of Rome, P.le Aldo Moro 5, 00185 Rome, Italy; § Biomolecular Mass Spectrometry and Proteomics, Bijvoet Center for Biomolecular Research, Utrecht Institute for Pharmaceutical Sciences, Utrecht University, Heidelberglaan 8, 3584 CS Utrecht, The Netherlands; ∥ Unit of Histology and Medical Embryology, Department of Anatomy, Histology, Forensic Medicine and Orthopedics, Sapienza University of Rome, Viale A. Scarpa 16, 00161 Rome, Italy; ⊥ Institute of inorganic Chemistry, Graz University of Technology, Stremayerg 6/IV, 8010 Graz, Austria; # NEST, Scuola Normale Superiore, Piazza San Silvestro 12, 56127 Pisa, Italy; ¶ Department of Experimental Medicine, University of Rome “Sapienza”, Viale Regina Elena 324, 00161 Rome, Italy; + Department of Clinical Sciences and Translational Medicine, University of Rome “Tor Vergata”, Via Montpellier 1, 00133 Rome, Italy; × Department of Medico-Surgical Sciences and Biotechnology, Sapienza University of Rome, Corso della Repubblica 79, 04100 Latina, Italy

**Keywords:** gene delivery systems, liposomes, lipoplexes, protein corona, immune cell interactions, stealth nanoparticles

## Abstract

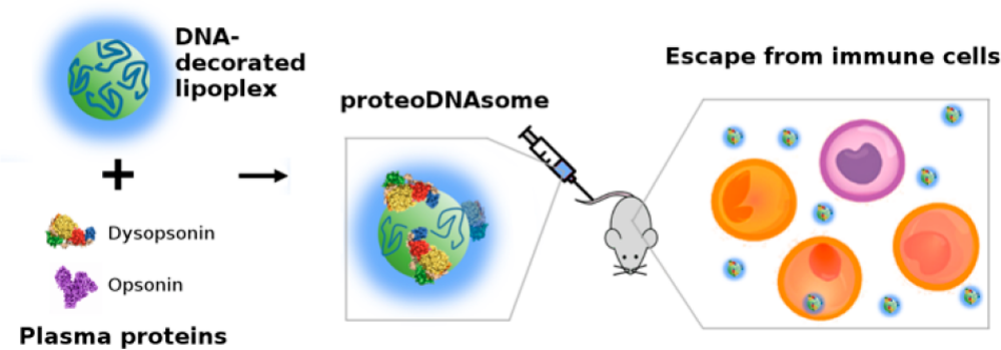

For several decades, surface grafted polyethylene glycol (PEG) has been a go-to strategy for preserving the synthetic identity of liposomes in physiological milieu and preventing clearance by immune cells. However, the limited clinical translation of PEGylated liposomes is mainly due to the protein corona formation and the subsequent modification of liposomes’ synthetic identity, which affects their interactions with immune cells and blood residency. Here we exploit the electric charge of DNA to generate unPEGylated liposome/DNA complexes that, upon exposure to human plasma, gets covered with an opsonin-deficient protein corona. The final product of the synthetic process is a biomimetic nanoparticle type covered by a proteonucleotidic corona, or “proteoDNAsome”, which maintains its synthetic identity *in vivo* and is able to slip past the immune system more efficiently than PEGylated liposomes. Accumulation of proteoDNAsomes in the spleen and the liver was lower than that of PEGylated systems. Our work highlights the importance of generating stable biomolecular coronas in the development of stealth unPEGylated particles, thus providing a connection between the biological behavior of particles *in vivo* and their synthetic identity.

The development of effective and safe gene-delivery systems (GDSs) is the main bottleneck toward successful clinical applications in different fields (e.g., cancer gene therapy, CGT).^1^ The GDSs currently at our disposal belong to two distinct groups: viral and nonviral ones. Viral GDSs exploit the intrinsic ability of viruses to transfer their genetic materials into host cells. Unfortunately, however, their clinical application has been hampered by several technical factors, including, among others, complex manipulation procedures, restrictions in size of the target gene, unavoidable host immune responses, and inflammatory toxicities.^2^

Nonviral GDSs, based for instance on the use of polymers, peptides, dendrimers, or liposomes, hold the potential to bypass the limitations of their viral counterparts. Among others, liposomes emerged as a preferred platform owing to their low cost, easy production, and no limitations in gene-payload size.^3^

Still, at least two main requisites must be implemented into an ideal nonviral, liposome-based GDS, which may appear as mutually exclusive: (i) capability to interact with biological membranes, thus favoring endosomal escape and cargo release within the target cells;^4^ (ii) stealth properties to prevent, at the same time, clearance by circulating blood cells (e.g., leukocytes).^5^

Requisite (i) is satisfied by cationic liposomes (CLs). CLs show the exquisite advantage of spontaneous interaction with negatively charged, membrane-associated proteoglycans, thus promoting robust cell-uptake of lipoplexes.^6^ Unfortunately, concerning requisite (ii), the positive charge of CLs elicits the adsorption of biomolecules from human blood, especially proteins, which in turn induce extensive aggregation and masking of the designed functionality.^2^ In particular, it was demonstrated that the peculiar adsorption of *opsonins* on the CL surface (*e.g.*, IgG complement proteins *etc.*) stimulates phagocytosis and rapid clearance of CLs from blood circulation. Of note, this effect is not completely prevented by CL-surface functionalization by poly(ethylene glycol) (PEG), as previously thought.^7^

On the other hand, lipoplexes made of anionic^8^ or zwitterionic^9^ lipids satisfy requisite (ii): they bind low amounts of proteins, leading to longer circulation times and satisfactory clearance profiles. Concerning point (i), however, intrinsic negative charge and consequent poor interaction with cell membranes of these systems are at the basis of their unsatisfactory performances. As result of these limitations, the field of lipoplex-mediated gene delivery has not advanced significantly over the past 2 decades.

To combine both the efficient interaction with cellular membranes and the stealth properties in a single lipoplex, here we propose a change of paradigm in the architecture of the GDS. From one side, DNA forms stable complexes with CLs that have the intrinsic capability to interact with biological membranes of target cells allowing efficient endosomal escape (*i.e.*, requisite “i”); On the other side, DNA is explored, due to its negative charge, as an alternative candidate for the construction of a stealth GDS (*i.e.*, requisite “ii”). In other words, upon exposure to biological fluid, DNA mainly recruits dysopsonins that provide the GDS with stealth properties against capture by immune cells.

Technically, the CLs used in our study were based on a multicomponent CL formulation consisting of DOTAP, DC-Chol, DOPC, and DOPE. This formulation (hereafter referred to as CL1) is a standard choice to maximize endosomal escape, even in hard-to-transfect cells.^10^ A second CL variant, hereafter termed CL2, was prepared by substituting DOPC with DOPG: according to previous findings, this change may lead to greater colloidal stability of the particles in serum/plasma but also increases membrane permeability to charged molecules (*e.g.*, DNA),^11^ thus leading to superior efficacy. Both CL formulations were incubated with functional plasmid DNA to form stable lipoplexes. As a first step, we thoroughly characterized chemical-physical properties of the lipoplexes. We could identify two distinct lipoplex structures with similar size but opposite zeta-potential: (i) positively charged complexes that contain most of the DNA in the vesicle core are hereinafter referred to as “plain lipoplexes” (PLs); and (ii) negatively charged complexes whose surface is fully decorated with DNA and are therefore termed “DNA-decorated lipoplexes” (DDLs). DDLs and PLs prepared from CL1 are hereafter indicated with DDL1 and PL1, while those prepared from CL2 are denoted with DDL2 and PL2, respectively. Based on previous experience, an adduct must be tested in terms of its ability to attract a protein biomolecular corona (BC) upon interaction with plasma, as BC directly modifies the original features of the adduct and shapes a biological identity that in turn affects its interaction with cells (*e.g.*, immune cells). Of note, exposure of DDLs to low human plasma (HP) concentrations (HP = 5%) promotes formation of an artificial protein corona deficient in typical opsonins (*e.g.*, complement proteins, immunoglobulins) as determined by proteomics experiments. This in turn leads to an unexpected but fundamental result: the absence of opsonins, in fact, can boost the stealth properties imparted by DNA by preventing the DDL2-protein complexes sequestration by blood cells. We explored the ability of these generated “*proteoDNAsomes*” to evade immune cells both *in vitro* and *in vivo* using PEGylated lipoplexes as a reference (Figure 1). We demonstrate the capacity of proteoDNAsomes to avoid capture by immune cells *in vitro* by using the human monocytic THP-1 and *ex vivo* leukocyte subpopulations derived from healthy donors. ProteoDNAsomes exhibited a peculiar capacity to evade the immune system *in vivo*. When administered to C57BL/6 mice, proteoDNAsomes avoided capture by populations of phagocytes in the blood (i.e., monocytes and neutrophils) and in the spleen (i.e., monocytes, neutrophils, and macrophages) (Figure 1) much more efficiently than state-of-the-art PEGylated lipoplexes.

**Figure 1:**
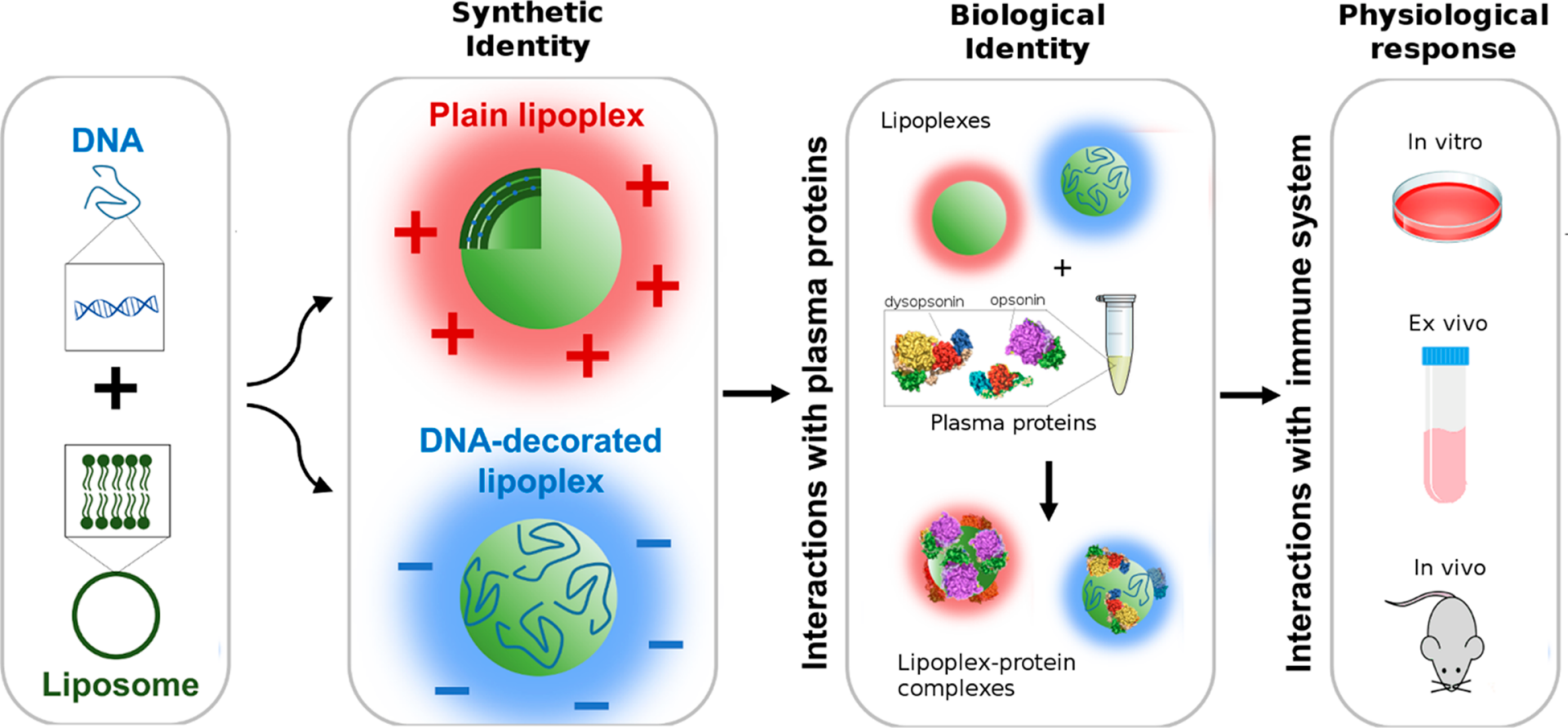
When liposome-DNA complexes (lipoplexes) with a precise synthetic identity are exposed to plasma proteins, they get covered by a protein corona that provides them with a biological identity.^12^ The biological identity of lipoplexes ultimately controls their physiological response (*e.g.*, interaction with the immune system). Interaction between lipoplexes and immune cells are tested using (i) human monocytic cell line THP-1 (*in vitro*), (ii) circulating leukocytes from human donors (*ex vivo*), and (iii) C57BL/6 mice (*in vivo*).

## Results

### DNA-Decorated Lipoplexes

**Figure 2:**
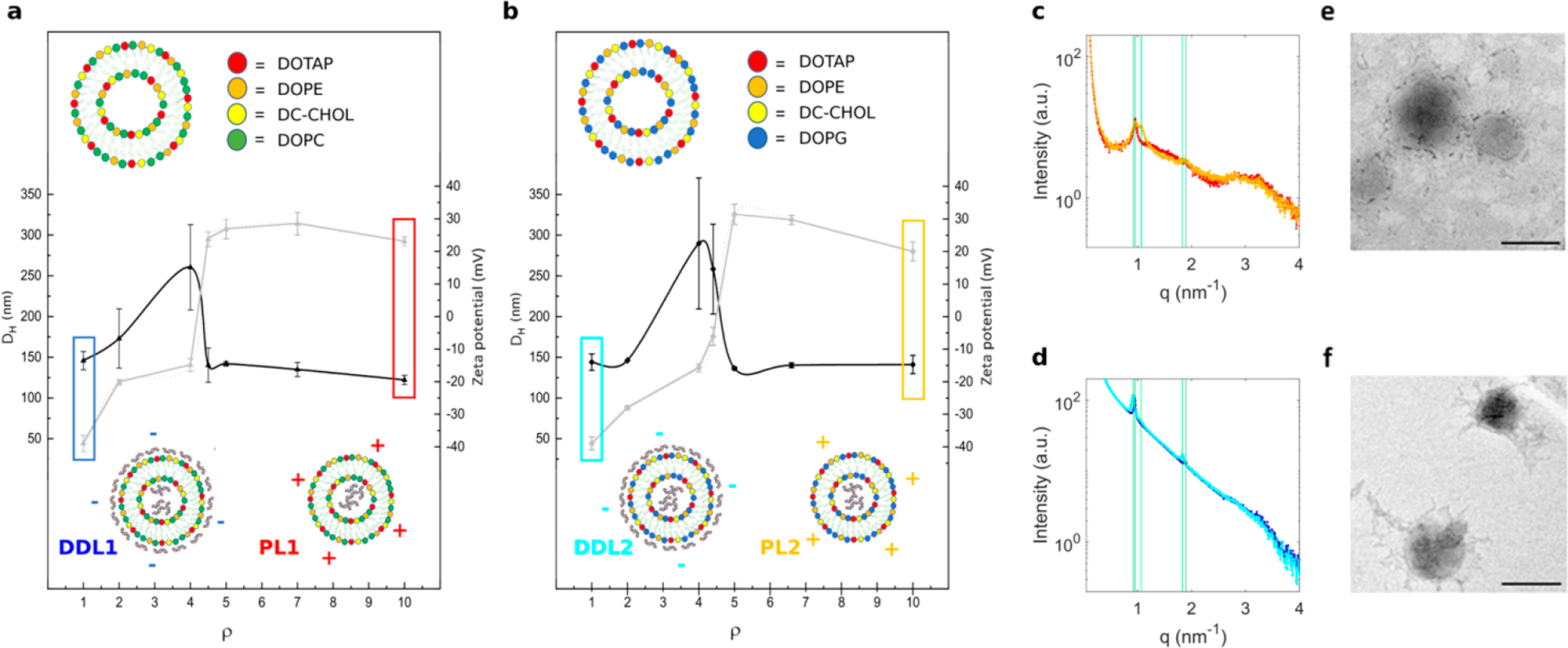
Characterization of lipoplexes as a function of the cationic lipid/DNA weight ratio. Size (black points) and zeta potential (gray points) of lipoplexes prepared from CL1 (panel a) and CL2 (panel b) as a function of the cationic lipid/DNA weight ratio, ρ. At ρ = 1, lipoplexes were small in size (*D*_H_ ≈ 150 nm) and negatively charged (zeta potential ≈ −40 mV) (blue and cyan boxes in panels a and b, respectively). This value was therefore identified as the proper cationic lipid/DNA weight ratio leading to DNA-decorated lipoplexes (DDLs) in line with the purpose of this study. On the other hand, we selected ρ = 10 as the lipid/DNA weight ratio leading to cationic plain lipoplexes (PLs, *i.e.*, whose surface is entirely lipidic) that were used as a control in the following experiments (red and gold boxes in panels a and b, respectively). Synchrotron small-angle X-ray scattering (SAXS) patterns of PLs (panel c) and DDLs (panel d). Color code: DDL1 (blue), DDL2 (cyan), PL1 (red), PL2 (gold). Vertical green lines indicate the position of the first- and second-order Bragg peaks arising from a multilamellar structure made of alternating lipid bilayers and DNA monolayers. Representative transmission electron microscopy (TEM) images of PLs (panel e) and DDLs (panel f). Both samples are visible nanosized, rounded-shaped vesicles. The presence of DNA filaments on the surface is detectable in DDLs. Bars correspond to 200 nm.

First, we performed dynamic light scattering (DLS), microelectrophoresis (ME), and synchrotron small-angle X-ray scattering (SAXS) experiments to explore the hydrodynamic diameter (*D*_H_), zeta potential (Zp), and nanostructure of CL1 and CL2. Results are listed in Figure S1 and Table S1. Both formulations were homogeneous, positively charged (Zp > 40 mV), and small in size (*D*_H_ < 140 nm). As a next step, we prepared lipoplexes by bulk mixing of DNA and CLs. Figure 2 shows the size (black line) and zeta-potential (gray line) of lipoplexes prepared from CL1 (panel a) and CL2 (panel b) as a function of the cationic lipid/DNA weight ratio, ρ. At ρ = 1, lipoplexes were small (*D*_H_ ≈ 150 nm), and negatively charged (Zp ≈ −40 mV). This value was therefore identified as the proper cationic lipid/DNA weight ratio leading to DDLs in line with the purpose of this study. On the other hand, we selected ρ = 10 as the lipid/DNA weight ratio leading to cationic plain lipoplexes that were used as a control in the following experiments. Comparing the zeta potential distributions of CLs and cationic lipoplexes, we concluded that the surface of PLs was poorly or not at all decorated with DNA molecules. Structures of PLs and DDLs were characterized by synchrotron SAXS. In Figure 2c,d, we compare the SAXS patterns of PLs and DDLs, respectively. In the SAXS patterns, two Bragg peaks were detected at *q*_001_ = 0.95 nm^–1^ and *q*_002_ = 0.190 nm^–1^. They arise from a multilamellar structure^13,14^ whose periodicity along the normal to the lipid bilayer, *d*, is the sum of the membrane thickness (*d*_B_) and the thickness of the water layer occupied by DNA molecules (*d*_W_): *d* = *d*_B_ + *d*_W_ = 2π/*q*_001_ = 6.61 nm. In the SAXS pattern of PL2, another broader Bragg peak was observed at *q*_001_ = 0.107 nm^–1^, corresponding to a lamellar spacing of *d* = 5.87 nm. According to the literature, this could indicate demixing into lipid-enriched phases.^15^

We also observe that SAXS patterns of DDLs exhibit a stronger diffuse scattering between Bragg peaks. As the solvent scattering background was properly subtracted (see the Materials and Methods), this large interpeak scattering contribution could be due to some excess DNA in solution. This is confirmed by TEM measurements (Figure 2 and Figure S2). The results shown in Figure S2 show that PLs are plain vesicles with a multilamellar inner structure, while DDLs are decorated with DNA protruding from the particle surface. Globally synchrotron SAXS and TEM outcomes suggest that the inner structure of lipoplexes is influenced by the cationic lipid/DNA charge ratio, while it is poorly dependent on the synthetic identity of pristine liposomes.

### Lipoplex-Protein Complexes

**Figure 3:**
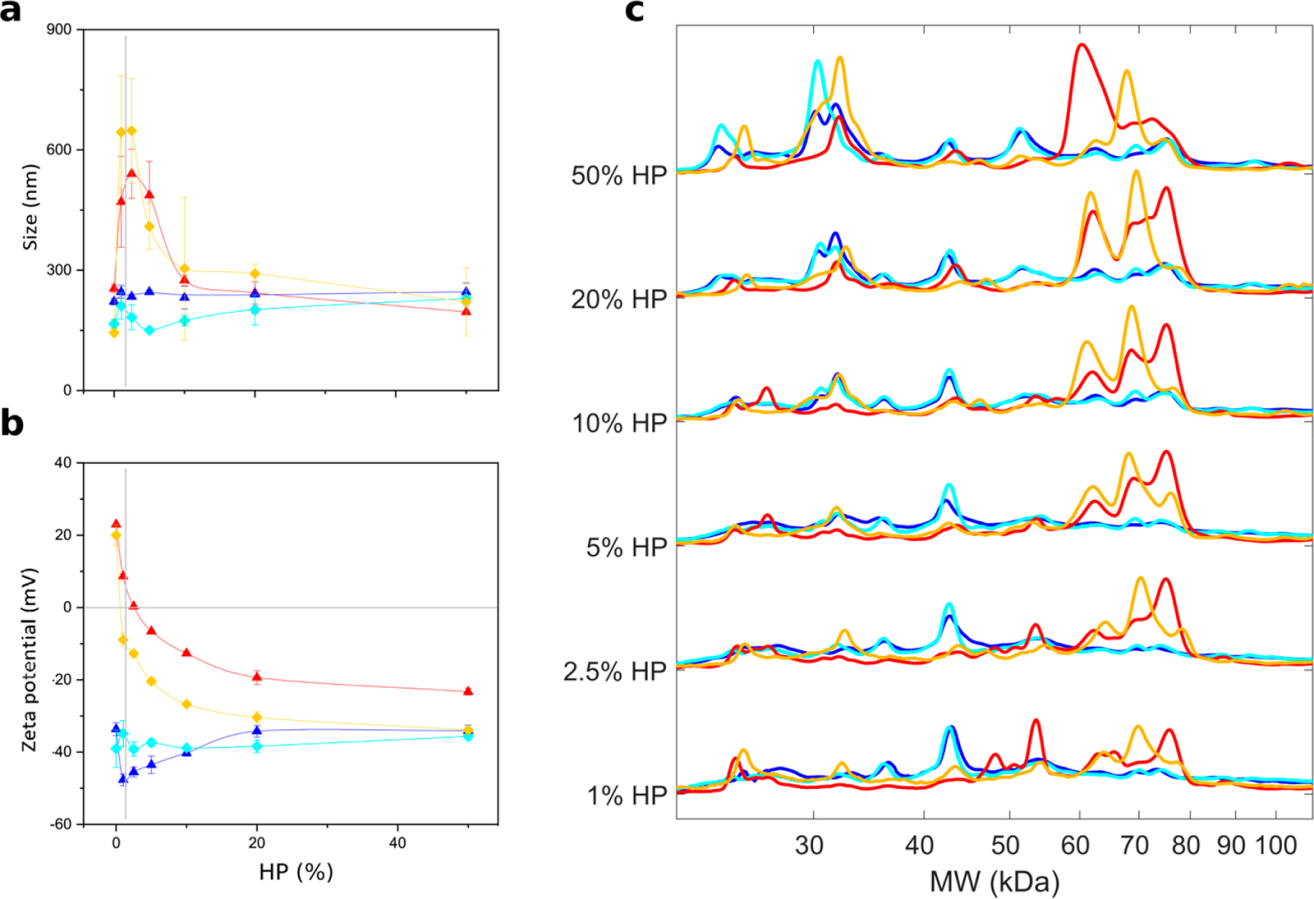
Characterization of lipoplex-protein complexes as a function of protein concentration. Size (panel a) and zeta potential (panel b) of lipoplex-protein complexes prepared from CL1 and CL2 as a function of human plasma (HP) percentage (%). As PLs are positively charged systems, PL-protein complexes exhibit the typical features of cationic-anionic assemblies, *i.e.*, re-entrant condensation and charge inversion at the isoelectric point. On the other side, the size of the DDL-protein complexes is poorly affected by HP and zeta potential tends to assume similar values. Vertical gray lines indicate the inversion point where PL-protein complexes undergo massive aggregation (panel a) caused by charge neutralization (panel b). (c) Molecular weight (MW) distributions of protein patterns bound to lipoplexes as a function of HP %. While protein patterns of DDL1- and DDL2-protein complexes were almost superimposable up to HP = 20%, those of PL1- and PL2-protein complexes were clearly distinguishable over the protein range especially between 45 and 80 kDa. Color code: DDL1 (blue), DDL2 (cyan), PL1 (red), PL2 (gold).

To promote the formation of lipoplex-protein complexes, we incubated PLs and DDLs in HP for 1-h at increasing protein concentrations. The size of the PL-protein complexes (Figure 3a) increases with increasing HP concentration, whereas the surface charge decreases (Figure 3b). In correspondence to the inversion of the zeta-potential (*i.e.*, 1% < HP < 5%), PL-protein complexes clustered in large aggregates due to their electronic neutrality. Large complexes form at the “isoelectric point”. When this happens, van der Waals interactions overcome the electrostatic repulsion and promote the formation of large neutrally charged assemblies. At higher HP concentrations, the electrostatic repulsion dominates and decreasing-size complexes form (re-entrant condensation).

The evolution profiles of size and zeta potential of PL-protein complexes suggest that anionic plasma proteins act as a molecular glue between distinct cationic lipoplexes and that protein amount is a crucial factor regulating the equilibrium physical-chemical properties of PL-protein complexes. These observations may have relevant implications, as clearance cells remove from the bloodstream objects larger than (roughly) 300 nm. At higher HP percentages, small size PL-protein complexes are observed. These findings agree with previous works obtained using other nanoparticle types.^16^ In contrast, the evolution profiles of size and zeta potential of DDL-protein complexes did not show appreciable variation in size and tended to assume similar zeta potentials at HP = 50%. Such an effect, termed “normalization” of the zeta potential has been previously observed for several types of nanomaterials.^17^

Among shaping factors, the protein concentration ratio plays a key role in determining the protein corona composition of NPs. To get an insight into the hard corona characteristics’ evolution, we isolated proteins from lipoplex-protein complexes and used one-dimensional (1D) sodium dodecyl sulfate-polyacrylamide gel electrophoresis (SDS-PAGE) to evaluate the molecular weight (MW) distributions of the adsorbed coronas (Figure S3). As Figure 3c clearly shows, PL1 and PL2 exhibited marked differences in their protein profiles at each protein concentration.^18^ Moreover, both the profiles evolved considerably. According to previous investigations,^19^ this indicates that the protein corona of PLs is made of both highly abundant low-affinity and poorly abundant competitive-binding proteins whose enrichment changes with the plasma concentration. Considering that PL1 and PL2 differ only in one of the four lipid species, our results confirm that the protein corona of lipid vesicles is considerably affected by the lipid composition.^20^ On the other hand, the corona of DDL1 and DDL2 was quite stable over the range of protein concentrations. This finding suggests that DNA decoration at the particle surface may have a dominant role in determining protein absorption to DDLs irrespective of the underneath lipid surface.

### Composition of the Lipoplex-Protein Corona

**Figure 4:**
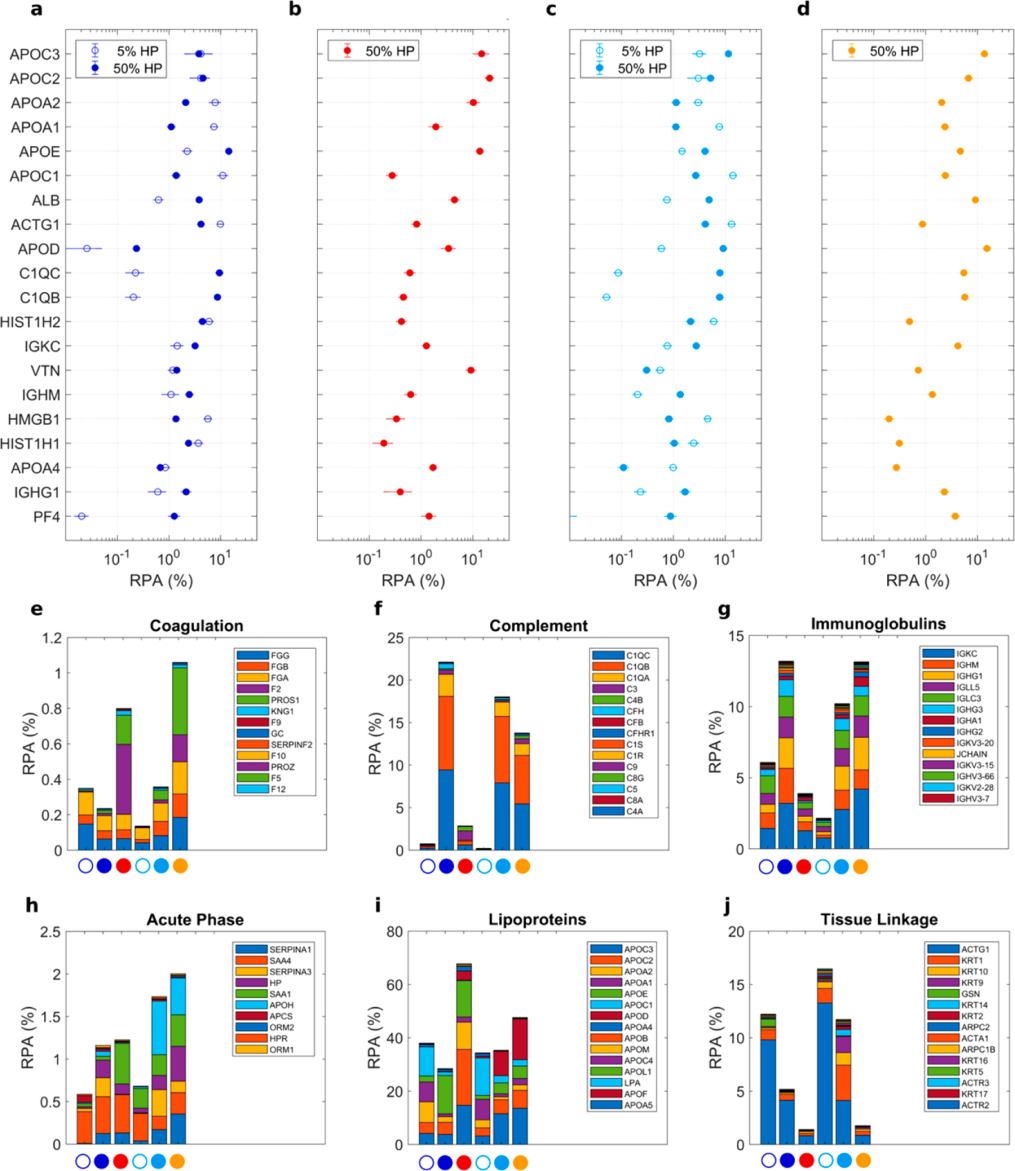
Bioinformatic classification of corona proteins. (a) Relative protein abundance (RPA) of the 20 most abundant plasma proteins (Top-20) identified in the coronas of DDL1 (blue), PL1 (red), DDL2 (cyan), and PL2 (gold) by quantitative nanoLC–MS/MS at the indicated human plasma (HP) concentrations. Plasma proteins were also grouped according to functional processes as reported in^34^ coagulation (e), complement (f), immunoglobulins (g), lipoproteins (e), acute phase (h), lipoproteins (i), and tissue leakage (f). Each value is the average of triplicates ± standard deviation within a single experiment.

Given the differences in the evolution of protein profiles as a function of protein concentration, we decided to investigate the composition of the protein corona to identify stealth and biosafe protein coatings for *in vivo* applications.^21^ According to the literature,^19^ we characterized protein corona at low (HP = 5%) and high (HP = 50%) protein concentrations, where the largest difference in physical-chemical properties of nanoparticle–protein complexes is typically found. However, as Figure 3a shows, the size of PL-protein complexes at low HP was too large (*D*_H_ > 500 nm) to be compatible with drug delivery purposes.^22,23^ These complexes were therefore excluded from further characterization. Table S2 lists all the plasma proteins identified in the coronas of DDLs and PLs by nano-LC-MS/MS. In Figure 4a–d, we report the relative protein abundance (RPA) of the top-20 proteins. MS/MS results show significant differences in the RPAs of PL1 and PL2. On the other hand, RPA of the top-20 proteins were similar for DDL1 and DDL2. This observation confirms SDS-PAGE findings demonstrating quantitatively that the lipid composition is one of the main factors shaping the protein corona of lipid vesicles, while the DNA layer adsorbed at the lipoplex surface makes the vesicle surface inaccessible. Finally, we observed that protein concentration has a marked effect on protein binding. In detail, APOA1, APOA2, APOC, ACTG1, and HMGB1 were more abundant when DDLs were exposed to low protein concentrations. On the other hand, binding of APOE, HAS, C1QC, C1QB, IGKG, and IGHM was favored by particle incubation at high protein concentrations. Proteins were also categorized according to the biological processes they are involved in.^24^ As Figure 4e–j clearly shows, enrichment in proteins involved in complement activation, coagulation, immune response, acute phase, and tissue leakage were appreciably affected by lipoplex characteristics. Experimental evidence supports the idea that decoding the composition of the nanoparticle–protein corona is fundamental to determine the stealth properties of NPs. However, hundreds of proteins are usually associated with NPs in different proportions, and understanding the role played by each protein component in regulating nanoparticle-cell interaction is a very difficult task. Most importantly, the protein corona is a complex “cloud” of biomolecules, and mapping protein location and exposure of functional epitopes is a big challenge.^25−28^ A longstanding popular view is that poor enrichment in opsonins is one of the most successful criteria in designing stealth protein coverages.^29,30^ Comparing therefore the enrichment in opsonins, we concluded that the protein coating of DDL2 at HP = 5% (total RPA of identified opsonins <3%) has the best stealth properties. A minor abundance of complement proteins and immunoglobulins was detected. We also observed that DDL2 was poorly enriched of fibrinogen that is an endogenous ligand for α_M_β_2_/Mac 1, the integrin receptor that is vital to regulate leukocyte function and trigger innate immunity *in vivo*. Languino *et al.* have demonstrated that binding of fibrinogen to vascular cell receptors promotes the adhesion of leukocytes to the endothelium and transendothelial migration, which are the initial events of immune inflammatory responses.^31^ More recently, Deng et al. demonstrated that fibrinogen, upon binding to some nanoparticle types undergoes denaturation, activates the integrin receptor Mac-1, and stimulates the NF-κB signaling pathway leading to the release of inflammatory cytokines in THP-1 cells.^32,33^

### Cellular Uptake by Monocytic THP-1 Cells

As known, nanoparticles interact with the innate immune system, including the complement system and different times of phagocyte populations. These cells display high phagocytic capacity and typically safeguard from foreign materials; however, recognition of nanoparticles as foreign may result in a multilevel immune response against the nanoparticles and eventually lead to a lack of therapeutic efficacy. Hence, we used flow cytometry to analyze the cellular uptake of DDL- and PL-protein complexes by the human monocytic THP-1 cell line, a spontaneously immortalized monocyte-like cell line derived from the peripheral blood of a childhood case of acute monocytic leukemia (M5 subtype).^35^

**Figure 5:**
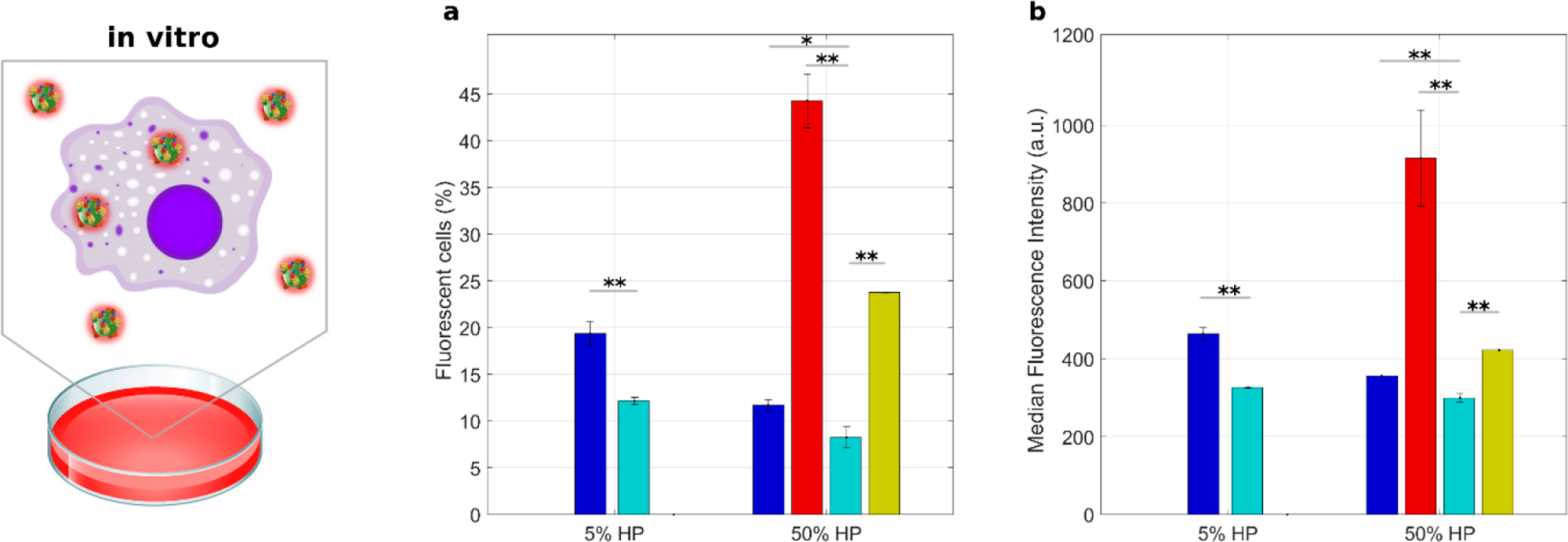
Sequestration of lipoplex-protein complexes by THP-1 cells *in vitro*. Cellular uptake of fluorescently labeled lipoplex-protein complexes by human monocyte THP-1 cells via flow cytometry evaluated in terms of percentage of fluorescent cells (a) and median fluorescent intensity (b). Complexes were prepared at low (*i.e.*, HP = 5%) and high (*i.e.*, HP = 50%) protein concentrations. Each value is the average of triplicate samples ± standard deviation within a single experiment. Statistical significance was evaluated by Student’s *t* test with respect to DDL2 lipoplexes. *p < 0.05, **p < 0.001, no asterisk means not significant. Color code: DDL1 (blue), DDL2 (cyan), PL1 (red), PL2 (gold).

At low protein concentrations (HP = 5%), the percentage of positive cells (Figure 5a) and median fluorescence intensity (Figure 5b) were low for both DDL1 and DDL2. At high protein concentration (HP = 50%), the cellular uptake of PLs was much higher than that of DDLs and dependent on lipid composition with PL1 > PL2. These results would point out the key role of the protein corona of PLs in determining capture by monocytes and consequent removal from the bloodstream *in vivo*. Second and foremost, the ability of DDLs to escape internalization by THP-1 could result in increased circulation time and consequently boosting the ability to reach therapeutic targets. To test this suggestion, we explored capture by circulating leukocytes in patients’ whole blood.

### Particle Sequestration by Circulating Leukocytes

**Figure 6:**
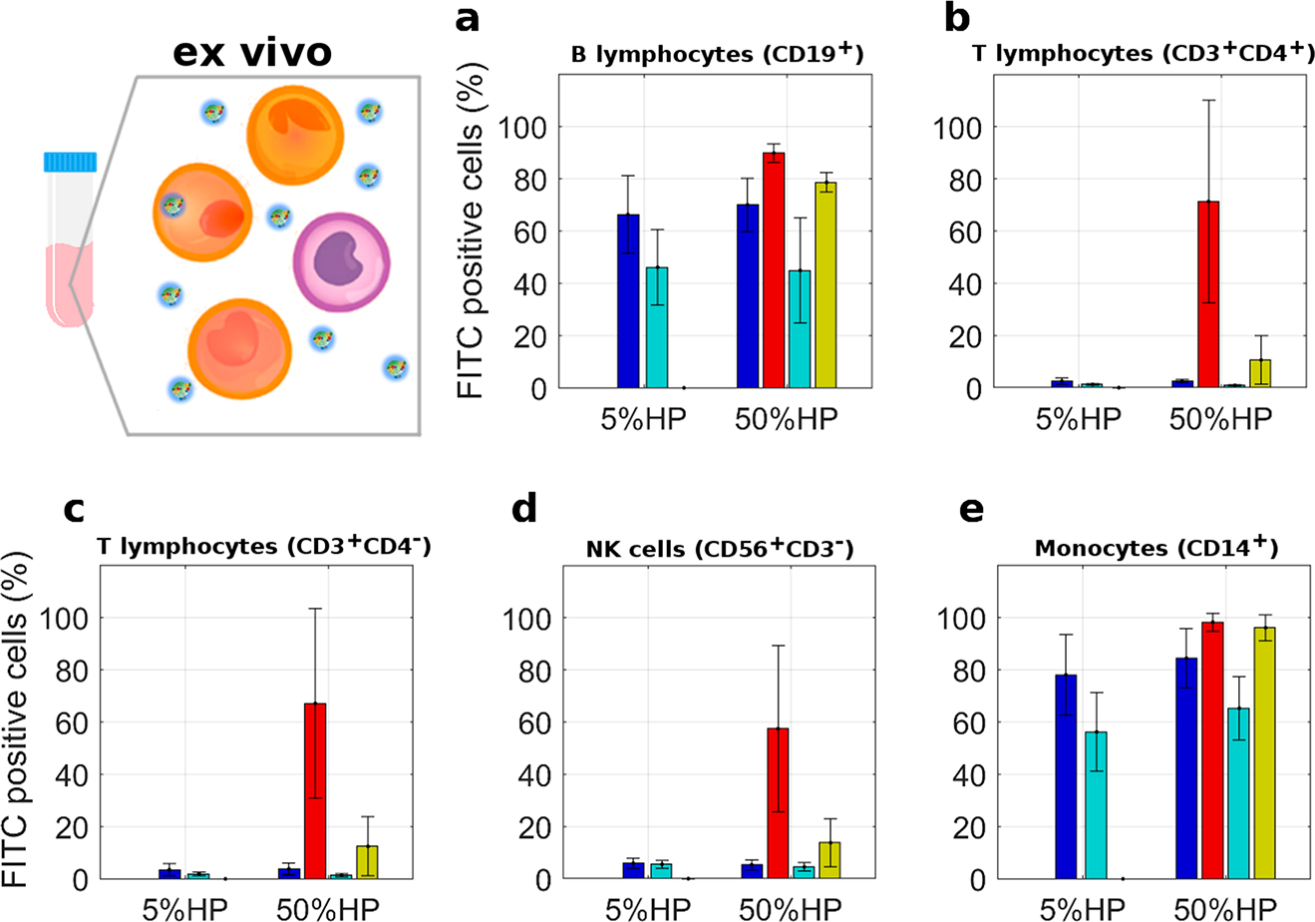
Leukocyte uptake of lipoplex-protein complexes. Complexes were prepared at low (5%) and high (50%) human plasma (HP) concentrations. Each value is the average of duplicate samples ± standard deviation within a single experiment. The fluorescence of internalized lipoplex-protein complexes was measured as the percentage of FITC-positive cells by gating on distinct leukocyte subpopulations as indicated. The gating strategy was obtained as explained in ref (37). Color code: DDL1 (blue), DDL2 (cyan), PL1 (red), PL2 (gold).

Uptake by circulating leukocytes represents part of the immune system which provides efficient surveillance for foreign pathogens. The introduction of nanoparticles via intravenous injection mimics the infection process and might trigger sequestration by leukocytes drastically affecting the particle biodistribution.^5^ To validate the trends found in the monocytic THP-1 cell lines, we used flow cytometry to evaluate the sequestration by both circulating monocytes and other distinct leukocyte subpopulations derived from peripheral blood mononuclear cells (PBMCs) of healthy donors (Figure 6). PBMCs have been widely used as an *in vitro* model to investigate immune system functions.^36^ As Figure 6 clearly shows, we observed that (i) leukocytes subpopulations of CD19^+^ (B lymphocytes) and CD14^+^ (monocytes) avidly internalized both PLs and DDLs, (ii) for each leukocyte subpopulation and HP percentage, cellular uptake of PLs was higher than that of DDLs and dependent on lipid composition (*i.e.*, PL1 > PL2), (iii) DDLs exhibit a peculiar ability to escape the sequestration by the other subpopulations. In particular, DDL2 at HP = 5% showed the lowest cellular uptake by leukocytes in patients’ whole blood.

Keeping in mind that the six tested formulations had, within the limits of the experimental error, the same synthetic identity (*i.e.*, size and zeta potential), we were able to conclude that the differences in uptake by leukocytes were not due to specific internalization mechanisms such as size-^4^ or charge-dependent^38^ cellular uptake. On the other hand, the considerable differences in the composition of the protein corona led us to conclude that the cellular internalization of lipoplex-protein complexes is largely regulated by receptor-mediated mechanisms. This reiterates the central role of the protein corona in mediating interactions between nanoparticle systems and cells in biological milieu.

Combining the indications arising from (i) physical-chemical characterization of nanoparticles, (ii) protein corona characterization, (iii) cellular uptake in THP-1, and (iv) capture by circulating leukocytes in whole blood, we were prompted to conclude that exposing DDL2 to low protein concentrations (*i.e.*, HP = 5%) leads to the formation of a stealth and biosafe protein coating. Protein-coated DDL2 was therefore identified as the most promising nanocarrier for further *in vivo* validation.

### Capture by Immune Cells *In Vivo*

**Figure 7:**
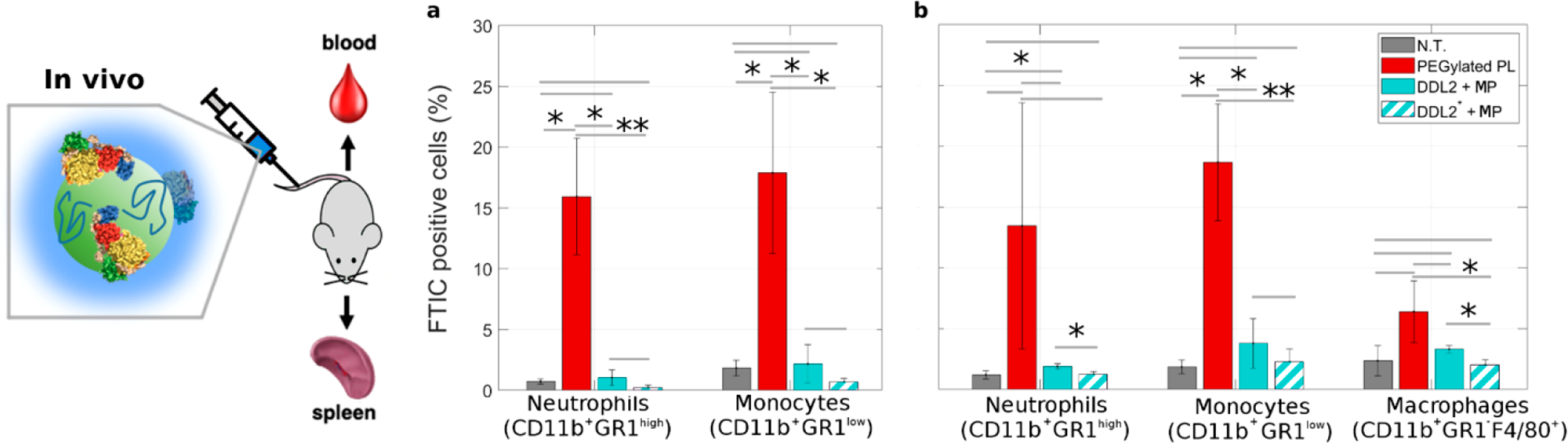
*In vivo* particle uptake by circulating and resident phagocytes. 8–10 weeks-old female C57BL/6 mice were i.v. injected with three types of lipoplexes, PEGylated plain lipoplexes (PEGylated PL), plasmid DNA-decorated lipoplexes coated with mouse plasma (MP) proteins (DDL2+MP) and 20-mer oligonucleotide-decorated lipoplexes coated with mouse plasma (MP) proteins (DDL2*+MP). In all the groups (*n* = 6 animals), capture by phagocytes was evaluated in the blood (panel a) and the spleen (panel b) as percentage of FITC-positive cells by flow cytometry gating on distinct populations as indicated. The gating strategy is illustrated in Figure S4. Naïve animals served as controls (*n* = 9–12). Data are expressed as the mean ± SD. Statistical significance was evaluated by Student’s *t* test. *p < 0.05, **p < 0.001, no asterisk means not significant.

*In vivo* animal experiments were performed to gain insight into the stealth properties of precoated DDL2. Tenzer et al.^34^ showed that the protein corona is established quickly (within minutes) and does not substantially change in composition over time. Moreover, Simon et al.^39^ have demonstrated that pre-formed protein corona remains stable even after nanoparticles are re-introduced to the plasma. Thus, particle capture by immune cells *in vivo* could not be biased by changes in corona composition after administration to mice. PEGylated PLs that have long been the gold standard of lipid-mediated *in vivo* gene delivery^40^ were used as a reference. PEG forms hydrogen bonds with water molecules leading to a hydration layer that, together with polymer chains, creates a steric barrier that attenuates protein binding. PEGylation reduces opsonization and macrophage uptake, thereby extending blood circulation times. However, an immune response against PEG hampers the promising role of PEGylation. Many reports have shown that systemic administration of PEGylated PLs induce robust anti-PEG IgM production that results in the accelerated blood clearance (ABC) phenomenon.^41^ Consequently, the development and evaluation of therapeutically useful unPEGylated formulations is urgently needed. We administered PEGylated PLs and precoated DDL2 to 8–10 weeks-old female C57BL/6 mice by intravenous venipuncture (i.v.) and evaluated particle capture by the main populations of phagocytes in the blood (*i.e.*, monocytes and neutrophils) and in the spleen (*i.e.*, monocytes, neutrophils, and macrophages). Our findings displayed in Figure 7 show that PEGylated PLs are massively captured by all the phagocyte populations both in the blood and in the spleen (the gating strategy is illustrated in Figure S4). In contrast, the phagocyte uptake of precoated DDL2 appears to be very low both in the blood and the spleen. In this work, we used plasmid DNA, as plasmids are used for several biomedical applications as tools to clone, transfer, and manipulate genes. However, to generalize our results, we prepared a variant of DDL2 using a commercial 20-mer oligonucleotide (in the following text indicated as “DDL2*”, all the details are found in Figure S5 in the Supporting Information). The uptake of precoated DDL2* by populations of phagocytes in the blood and in the spleen is very low (Figure 7). This observation let us to conclude that the DNA size does not affect the stealth properties of “proteoDNAsomes”. The *in vivo* stealth properties of precoated DDL2 and DDL2* are significant considering that clinically approved PEGylated liposomal formulations cannot completely prevent opsonin adsorption and that the PEG layer can interfere with the interaction of particles with cells *in vivo* in a rather unpredictable manner depending on the molecular weight and branching of the PEG molecules.^42^ For example, Hadjidemetriou et al. investigated the formation of protein coronas on clinically used PEGylated liposomal doxorubicin (DOXIL) nanoparticles following their intravenous administration in CD-1 mice^43^ and humans.^44^ They found that PEGylated liposomes *in vivo* become heavily coated with opsonins, and the protein corona has an impact on cellular internalization by immune cells, which helped to understand the poor clinical success of these formulations.

**Figure 8:**
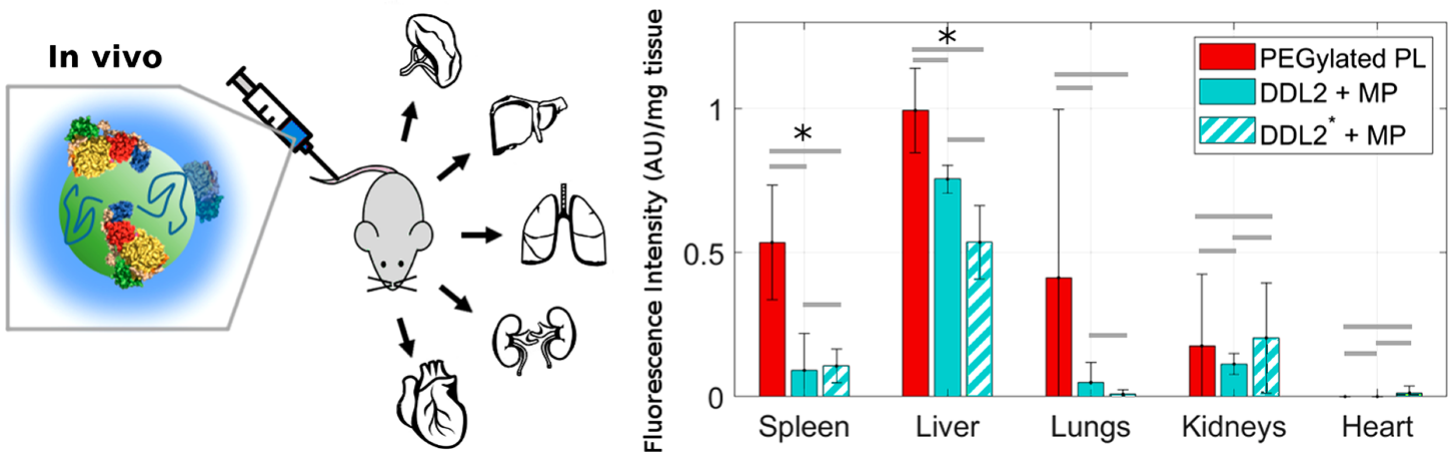
Biodistribution of lipoplexes in C57BL/6 mice. Distribution of particles in the spleen, liver, lungs, kidneys, and heart of C57BL/6 mice was evaluated by measuring the fluorescence intensity per mg of tissue. Data are expressed as the mean ± SD. Statistical significance was evaluated by Student’s *t* test. *p < 0.05, **p < 0.001, no asterisk means not significant.

As a further investigation, we carried out a biodistribution study by injecting mice with PEGylated PLs, precoated DDL2, and precoated DDL2* and then sacrificing the mice at 1-h after injection. Particle distribution was evaluated by measuring fluorescence in homogenates of the lymphoid (spleen) and the nonlymphoid (liver, lungs, kidneys, and heart) organs. Biodistribution results are reported in Figure 8. Lymphoid organs showed significantly lower levels of precoated DDL2 and precoated DDL2* than those of PEGylated PL. In the lung, accumulation of PEGylated PL was higher than those of precoated DDL2 and DDL2*, but experimental variability limited the statistically significant modifications. The levels of PEGylated PL, precoated DDL2, and precoated DDL2* were low in the kidneys and in the heart and differences among formulations were not statistically significant. *In vivo* particle uptake by circulating and resident phagocytes and biodistribution of particles demonstrated that precoating DNA-decorated lipoplexes is a powerful strategy to increase systemic circulation.

Many studies have explored the intracellular signaling pathways activated upon the immune response to GDSs. Previous investigations^45^ have demonstrated that toll-like receptors (TLRs) are involved in this process and can affect the safety and effectiveness of GDSs. Among them, TLR9 is an important receptor expressed in immune system cells including dendritic cells, macrophages, natural killer cells, and other antigen presenting cells. TLR9 binds DNA that is rich in unmethylated CpG-DNA motifs and triggers signaling cascades that lead to a pro-inflammatory cytokine response. As a last step of our investigation, we asked whether PLs and DDLs could engage TLR9. To this aim, we used a 293-derived reporter cell line engineered to coexpress TLR9 along with an NF-kB-driven luciferase reporter. As shown in Figure S6, exposure to specific TLR9 agonists induced luciferase activity in the reporter cell line, whereas no effect was observed in the presence of distinct lipoplexes, thus suggesting that the DNA sequence is crucial to activate TLR9.

## Conclusions

Due to their positive net charge that promotes association with target cells, CLs have been the preferred template for gene delivery applications so far. Despite these efforts, however, their clinical application is still limited. A major limitation is that, upon exposure to a biological environment, CLs unavoidably elicit the adsorption of biomolecules from human blood, especially proteins, which in turn alter their synthetic identity and influence their final fate and efficacy in the target tissues. A major detrimental effect, for instance, is imposed by spontaneous absorption on the CL surface of opsonins (e.g., IgG, CO3b *etc.*), which promote cell association, endocytosis, and clearing by macrophages and other phagocytes, thus severely hampering the final efficacy of the gene-delivery adduct.^46,47^ In response to these limitations, two main research efforts can be identified in the literature. On one side, researchers developed strategies to abolish protein adsorption. Unfortunately, such nonspecific process cannot be eliminated by grafting polymers at the particle surface (*e.g.*, by PEG).^48,49^

On the other side, researchers attempted to control the composition of the adsorbed proteins to nanoparticles. For instance, the engineering of a preformed protein corona enriched in proteins with null or negligible affinity for the clearance-deputed cellular system became one of the most promising design efforts. Precoating nanoparticles with single protein(s) (*e.g.*, human serum albumin,^50^ transferrin,^51^ vitronectin) could enable controlled cellular interactions with biological systems. However, when exposed to a physiological environment rich in thousands of plasma proteins (e.g., serum/plasma), it is still possible for the particle to become coated with other proteins by the formation of a double or multiple layer.^52^ Alternatively, the idea was to modify the surface of the nanoparticle in such a way that the spontaneously adsorbed protein corona in the physiological environment gets naturally enriched in components of interest, with the undoubted advantage of generating a protein corona that will remain stable in the physiological environment. For instance, some of us designed a cationic lipid/DNA complex that, upon exposure to HP, gets coated with vitronectin-enriched protein corona with the selective ability to target cancer cells expressing high levels of the vitronectin α_ν_β_3_ integrin receptor.^53^ Unfortunately, this approach, at present, does not allow fine-tuning of the composition of the corona. Also, many adjustments in the synthetic identity and incubation process (*e.g.*, exposure time, protein concentration, temperature) may be necessary to achieve the desired “biological identity” (*i.e.*, size, zeta potential, and protein corona composition).^37^

**Figure 9:**
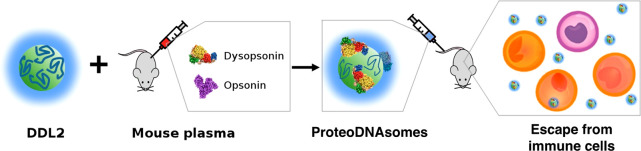
Exposing DNA-decorated lipoplexes to plasma proteins collected form C57BL/6 mice leads to formation of lipoplexes enriched with an opsonin-deficient, dysopsonin-enriched protein corona. The final product of the formulation process is a type of lipoplexes covered by a proteonuclotidic corona, or “*proteoDNAsome*”, with more pronounced ability to evade capture by immune cells *in vivo* than PEGylated lipoplexes.

In this work, we have exploited the electric charge of DNA to generate lipoplexes with a negatively charged surface which, upon exposure to human plasma, gets spontaneously covered with an outer biomolecular layer deficient in opsonins and enriched in dys-opsonins (Figure 9). The result is a peculiar biomolecular corona which can significantly influence the interaction of the whole adduct with immune cells both *in vitro* and *in vivo*. To summarize, the final product of the synthetic process is a species of lipoplexes covered by a proteonuclotidic corona, or “*proteoDNAsome*”, which are endowed with peculiar stealth properties. Our results demonstrate how the synthetic identity of nanocarriers can be engineered by fine-tuning their structural parameters (*i.e.*, biomolecular corona, DNA layer, lipoplex) to shape their biological identity and thus regulate their immune response.

## Material and Methods

### Chemicals

Cationic lipids 1,2-dioleoyl-3-trimethylammonium-propane (DOTAP) and (3β-[*N*-(*N*′,*N*′-dimethylaminoethane)-carbamoyl])-cholesterol (DC-Chol), zwitterionic lipids dioleoylphosphocholine (DOPC) and dioleoylphosphatidylethanolamine (DOPE), and anionic 1,2-dioleoyl-sn-glycero-3-phospho-(1′-rac-glycerol) (DOPG) were purchased from Avanti Polar Lipids (Alabaster, AL) as previously reported.^7^ Lipids were used without additional modification. Human plasma (HP) was purchased from Sigma-Aldrich, Inc. (Merk KGaA, Darmstadt, Germany). Plasmid DNA (pmirGLO plasmid vector) was purchased from Promega Corporation (Madison, WI). Oligonucleotide ODN 2243 was purchased from InvivoGen (Toulouse, France). Cy3-labelled plasmid DNA (Label IT Plasmid Delivery Control Cy3) was purchased from Mirus Bio (Madison, WI). Texas red-DOPE (Life Technologies, Carlsbad, CA) was purchased from Sigma-Aldrich, Inc. (Merk KGaA, Darmstadt, Germany), as previously reported.^54^

### Liposome and Lipoplex Preparation

We designed two multicomponent liposomal formulations. The first formulation (CL_1_) was made of the cationic lipids DOTAP and DC-Chol and the zwitterionic lipids DOPC and DOPE. The second formulation (CL_2_) was prepared by replacing DOPC with anionic DOPG. Both cationic liposomes were prepared at the desired molar ratio (DOTAP/DC-Chol/DOPC/DOPE = 1:1:1:1 for CL_1_ and DOTAP/DC-Chol/DOPG/DOPE = 1:1:1:1 for CL_2_). For *in vivo* experiments, formulations were labeled with Texas red-DOPE (fluorescent lipid/total lipid molar ratio = 4/1000), as previously reported.^54^ Each lipid was dissolved in chloroform, and the solvent was evaporated under a vacuum for 2 h. Lipid films were hydrated to obtain a final lipid concentration of 1 mg/mL with ultrapure water and stored at 4 °C. The obtained liposomal solutions were extruded 20 times through a 0.1 μm polycarbonate carbonate filter with the Avanti Mini-Extruder (Avanti Polar Lipids, Alabaster, AL), as previously reported.^37^ Plain and DNA-decorated lipoplexes (PLs and DDLs) were formed by mixing proper amounts of aqueous solutions of liposomes (1 mg/mL) and plasmid DNA (1 mg/mL) or Cy3 (500 ng/μL). The final solutions were left for 20 min at room temperature prior to performing the experiments. For size, zeta-potential, and SDS-PAGE, proteomics non-labelled plasmid DNA was used. For *in vitro*, *ex vivo*, and *in vivo* flow cytometry experiments, Cy3-labeled plasmid DNA was employed.

### Lipoplex-Protein Complexes

Lipoplex-protein complexes were prepared by incubating for 1-h proper amounts of lipoplexes in the following plasma concentrations: 1%, 2.5%, 5%, 10%, 20%, and 50%. For DLS and biological experiments, the resulting solution were characterized without further treatment. For SDS-PAGE and proteomics, lipoplexes-protein complexes were isolated by centrifugation for 15 min at 14 000 rpm. The pellets were washed three times with PBS to remove unbound proteins obtaining the “hard corona” (further details can be found in ref (55)).

### Size and Zeta Potential Experiments

Size and zeta potential measurements were carried out using a Zetasizer Nano ZS (Malvern, U.K.) at 25 °C. To perform size and zeta-potential experiments, sample solutions were diluted with distilled water (final volume = 1 mL), as previously reported.^37^ Results are given as mean ± standard deviation of three replicates.

### Synchrotron Small-Angle X-ray Scattering Experiments

SAXS measurements were carried out at the Austrian SAXS beamline at the synchrotron light source of Elettra (Trieste, Italy). SAXS scans were recorded in the *q*-ranges from 0.05 nm^–1^ to 5 nm^–1^ with a resolution of 5 × 10 nm^–3^. Calibration of detectors was performed with silver behenate powder (*d*-spacing = 5.838 nm). The sample was held in a 1 mm glass capillary (Hilgenberg, Malsfeld, Germany), and the measurements were executed at room temperature, as previously reported.^37^

### Transmission Electron Microscopy Experiments

Samples (10 μL) were dropped on formvar-carbon-coated copper grids (EMS, PA) and allowed to adsorb for 5 min. The resulting film was stained with a 2% uranyl acetate solution for 1 min at room temperature. The excess of staining solution was adsorbed by the filter paper. Grids were air-dried for 1 h before imaging with TEM Morgagni 268D (Philips, The Netherlands), as previously reported.^37^

### 1D SDS-PAGE Experiments

Lipoplex-protein complexes were resuspended in 40 μL of Laemmli Loading buffer 1× and boiled for 10 min at 100 °C. Each sample was loaded on a gradient polyacrylamide gel stain free (4–20 % TGX precast gels, BioRad) and run at 100 V for about 150 min. Finally, gel images were acquired with a ChemiDoc gel imaging system (Bio-Rad, CA) and processed by means of custom MatLab scripts (MathWorks, Natick, MA), as previously reported.^55^

### Proteomics

To perform nanoliquid chromatography-tandem mass spectrometry (nanoLC-MS/MS) experiments, lipoplex-protein complex pellets were resuspended in 40 μL of 8 mol L^–1^ urea and 50 mmol L^–1^ NH_4_HCO_3_ (pH = 7.8). Afterwards, protein solution was treated and prepared for the liquid chromatograph-mass spectrometer through a procedure accurately described elsewhere.^25^

### Cell Culture

Human monocyte cell line THP-1 cells were maintained in the RPMI-1640 (Gibco, Carlsbad, CA) medium supplemented with 2 mM l-glutamine, 100 IU/mL penicillin/streptomycin, and 10% fetal bovine serum until use.

### Flow Cytometry

To investigate the cellular uptake of lipoplex-protein complexes in the THP-1 cell line, lipoplexes were prepared with Cy3-labeled DNA. THP-1 cells were plated at 500 000 cells mL^–1^ in 12-well dishes. After the treatment, cells were washed with cold PBS and then run on a FACS Canto (BD Biosciences, San Jose, CA). Cells were gated using forward versus side scatter to exclude debris and then analyzed for the specific emission. The data were analyzed using FlowJo software (FlowJo LLC data analysis software, Ashland, OR), as previously reported.^37^ Full experimental details can be found in the supporting Excel file.

### Particle Sequestration from Circulating Leukocytes

PBMCs were isolated from peripheral blood of healthy donors by Ficoll–Hypaque gradient centrifugation. Cells were plated at 1 × 10^6^ cells mL^–1^ and then were incubated with lipoplexes for 1 h at 37 °C. After treatment with PL- and DDL-protein complexes, cells were washed with PBS and then labelled with the following diluted antibodies: anti-CD3/BV510 (catalog no. 564713, dilution 1:50), CD56/BV421 (catalog no. 562751, dilution 1:50), anti-CD4/APC (catalog no. 555349, dilution 1:10), anti-CD14/PerCP (catalog no. 340585, dilution 1:50), anti-CD45/allophycocyanin-H7 (catalog no. 560178, dilution 1:50), and anti-CD19/PE-Cy7 (anti-CD3/BV510 (catalog no. 564713557835, dilution 1:100) all from BD Bioscience. The fluorescence of the internalized liposomes was evaluated by immunofluorescence and FACS analysis using a FACSCanto (BD Biosciences, San Jose, CA) and measured as the percentage of positive cells by gating on distinct leukocyte subpopulations. The data analysis was performed using the FlowJo program, as previously reported.^37^ Full experimental details can be found in the supporting Excel file.

### *In Vivo* Animal Experiments

To investigate cellular uptake of nanoparticles *in vivo*, 200 μL of (i) PEGylated PL, (ii) protein coated-DDL2, and (iii) protein-coated DDL2* were injected by i.v. in 8–10 weeks-old female C57BL/6 mice (Charles River, *n* = 6 mice per group; (200 μL/each mouse)). The same volume of PBS was injected in the control group. At 1-h after injection, mice were sacrificed followed by cardiac puncture to collect blood, further added to the heparin tubes. Freshly isolated cells from the spleen and blood were prepared and stained, as previously described.^56^ Briefly, after lysis of red blood cells, 2 × 10^6^ of splenocytes or 50 μL of peripheral blood mononuclear cells (PBMCs) were washed and resuspended in staining buffer (PBS without Ca^2+^ Mg^2+^, BSA 0.5%, EDTA 2 mM, and NaN_3_ 0.025%). After 10 mins of incubation with anti-CD16/32 (clone 24G2), cells were stained with the indicated mAbs (anti-F480/FITC, anti-CD11b/BV421, and anti-GR1/APC) for 25 min at 4 °C. The gating strategy is reported in Figure S4. Samples were analyzed by flow cytometry using a FACSCanto II (BD Biosciences, San Jose, CA), and data analysis was performed using the FlowJo program. For biodistribution analysis, spleen, liver, lungs, kidneys, and heart were collected and kept in PBS on ice. Solid organs were weighed and homogenized, and then PBS was added to each tube to bring it to a specific volume, 2× PBS (weight/volume). Organ homogenates (50 μL) were pipetted into a 96-well plate and then read using a fluorescent plate reader (BD Biosciences). The fluorescence values per mg of tissue were calculated. All animal experiments were approved by local ethic authorities and conducted in accordance with Italian Governing Law (D.lgs 26/2014; Prot. No. 03/2013). Animals were housed in the Institute’s Animal Care Facilities, which meet international standards and were checked regularly by a certified veterinarian responsible for health monitoring, animal welfare supervision, and revision of experimental protocols and procedures. Full experimental details can be found in the supporting Excel file.

### NF-kB Luciferase Reporter Assay

TLR-specific activation assays were performed using human embryonic kidney 293 (HEK293) cells expressing luciferase under control of the NF-kB promoter and stably transfected with TLR9 (TLR9-HEK293) or without TLR9 as a negative control (Luc-HEK293) purchased from InvivoGen (Toulouse, France). HEK293-transfected cells were maintained in DMEM supplemented with 4.5 g/L glucose and 10% FBS, 1% penicillin/streptomycin solution (Invitrogen), 5 μg/mL of puromycin (Sigma-Aldrich, St. Louis, MO) and 5 μg/mL of blasticidin (InvivoGen). For the NF-kB luciferase assay, 40 000 cells/well were seeded in 100 μL of complete DMEM without antibiotics in 96-well plates and incubated for 18 h at 37 °C. After this incubation, the medium was removed, cells were washed with PBS, and 200 μL of DMEM without FBS were added. Cells were incubated with suitable amounts of PL1, PL2, DDL1, and DDL2 and left for 5 h. As a positive control, a specific TLR9 agonist was used (*i.e.*, 50 μM ODN2216 from InvivoGen). After incubation, supernatants were aspired from each well, cells were washed with PBS and then were lysed for 15 min at room temperature using 50 μL/well of 1:5 diluted “passive lysis buffer” (Promega, Madison WI). The protein concentration was evaluated by Bio-Rad Protein Assay. In total, 3 μg of total proteins for each sample were diluted in 50 μL of PBS, and 50 μL of luciferase assay substrate (Promega, Madison WI) was added. Emitted light was immediately quantified using a luminometer Glo-Max-Multi Detection System (Promega, Madison WI). Full experimental details can be found in the supporting Excel file.

### Statistical Analysis

For all the experiments, results are reported as mean ± standard deviation (error bars in the charts). The number of replicates varied among experiments, as follows: *n* = 3 for size, zeta-potential, synchrotron SAXS measurements, and proteomics experiments; *n* = 2 for 1D SDS-PAGE and FACS analysis on the THP-1 cell line. For PBMCs experiments, *n* = 5. For *in vivo* experiments, *n* = 18 mice were sacrificed.

### Minimum Information Reporting in Bio–Nano Experimental Literature (MIRIBEL)

The studies conducted herein, including physical-chemical characterization, biological studies, and all the experimental details, conform to the MIRIBEL reporting standard for bio–nano research, and we incorporate a companion checklist of this information as Supplementary Table S3.

## Supporting Information Available

Supporting Excel file, scheme of the *in vitro* and *in vivo* experiments (XLSX)
Figure S1, size, zeta potential, synchrotron SAXS patterns, and the corresponding computed electron density profiles of cationic liposomes CL1 and CL2; Table S1, DLS and synchrotron SAXS parameters for cationic liposomes CL1 and CL2; Figure S2, representative transmission electron microscopy (TEM) images of plain and DNA-decorated lipoplexes; Figure S3, protein corona analysis of plain and DNA-decorated lipoplexes at increasing amounts of human plasma (HP); Table S2, lists of plasma proteins identified in the coronas of DDLs and PLs by nano-LC-MS/MS; Figure S4, gating strategy of mouse phagocyte populations; Figure S5, characterization of CL2/oligonucleotide (ON) lipoplexes as a function of the cationic lipid/ON weight ratio, ρ; Figure S6, luciferase activity in HEK293/Luc reporter cells transfected or not with TLR9 and incubated for 5 h with PL1, PL2, DDL1, DDL2, or a specific TLR9 agonist; and Supplementary Table S3, companion checklist about Minimum Information Reporting in Bio–Nano Experimental Literature (MIRIBEL) (PDF)

## Notes

Data Availability: The data sets generated during and/or analyzed during the current study are available from the corresponding author by reasonable request.

## Notes

Code Availability: Custom codes used for SDS-PAGE are available from the corresponding author by request.
