# Supplementary material for: Opsonin-Deficient Nucleoproteic Corona Endows UnPEGylated Liposomes with Stealth Properties In Vivo
Source: ACS Nano. 2022 Jan 18;16(2):2088–100. doi: 10.1021/acsnano.1c07687 (PMC8867903; doi:10.1021/acsnano.1c07687)
Supplement: Supplementary file 2 — nn1c07687_si_002.pdf [file nn1c07687_si_002.pdf]

Supporting Information

Opsonin-deficient nucleoproteic corona endows unPEGylated liposomes with stealth properties in vivo

Francesca Giulimondi,[1#] Elisabetta Vulpis,[1#] Luca Digiacomo,[1] Maria Valeria Giuli,[1] Angelica Mancusi,[1] Anna Laura Capriotti,[2] Aldo Laganà,[2] Andrea Cerrato,[2] Riccardo Zenezini Chiozzi,[3] Carmine Nicoletti,[4] Heinz Amenitsch,[5] Francesco Cardarelli,[6] Laura Masuelli,[7] Roberto Bei,[8] Isabella Screpanti,[1] Daniela Pozzi,[1] Alessandra Zingoni,[1] Saula Checquolo,[9] Giulio Caracciolo[1*]*

[1]Department of Molecular Medicine, Sapienza University of Rome, Viale Regina Elena 291, 00161 Rome, Italy

[2]Department of Chemistry, Sapienza University of Rome, P.le Aldo Moro 5, 00185 Rome, Italy

[3]Biomolecular Mass Spectrometry and Proteomics, Bijvoet Center for Biomolecular Research, Utrecht Institute for Pharmaceutical Sciences Utrecht University, Heidelberglaan 8, 3584 CS Utrecht, The Netherlands

[4]Unit of Histology and Medical Embryology, Department of Anatomy, Histology, Forensic Medicine and Orthopedics, Sapienza University of Rome, Via. A. Scarpa 16, 00161 Rome, Italy

[5]Institute of inorganic Chemistry, Graz University of Technology, Stremayerg 6/IV, 8010 Graz, Austria

[6]NEST, Scuola Normale Superiore, Piazza San Silvestro 12, 56127 Pisa, Italy

[7]Department of Experimental Medicine, University of Rome "Sapienza", Viale Regina Elena 324, 00161 Rome, Italy

[8]Department of Clinical Sciences and Translational Medicine, University of Rome "Tor Vergata", Via Montpellier 1, 00133 Rome Italy

[9]Department of Medico-Surgical Sciences and Biotechnology, Sapienza University of Rome, Corso della Repubblica 79, 04100 Latina, Italy

[#] These authors contributed equally

[*]Correspondence: saula.checquolo@uniroma1.it (S.C.); giulio.caracciolo@uniroma1.it (G.C.)

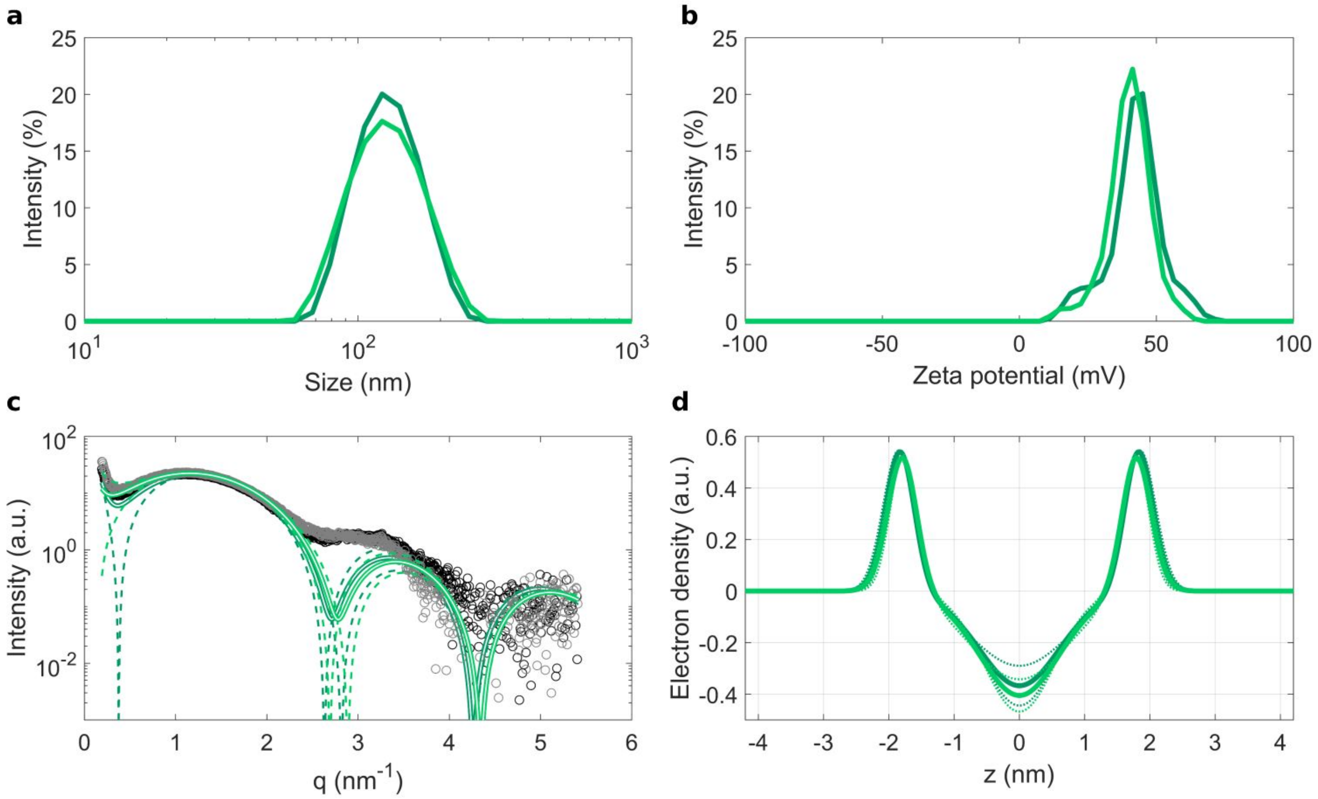

Figure S1. Characterization of CL1 (light green) and CL2 (dark green). (a) Size and (b) zeta potential distributions, (c) synchrotron SAXS patterns with the corresponding fitting curves and (d) computed electron density profiles. Structural parameters of lipid bilayers are calculated according to [Caracciolo et al. Langmuir 2006, 22, 4267-4273] and are reported in Table S1.

Table S1. DLS and synchrotron SAXS parameters measurements for CL1 and CL2. The parameters of the Gaussians fitting the electron density profiles reported in Figure S1d are: ρ_h (electron density of polar headgroups); σ_h (width of the positive Gaussian representing lipid headgroups); ρ_c (electron density profile of hydrocarbon chains); σ_c (width of the negative Gaussian representing hydrophobic core); z_h (distance between polar headgrouops of lipid monolayers).

	**CL1**	**CL2**
**D_H (nm)**	137	132
**P.d.I.**	0.14	0.11
**Zeta potential (mV)**	41.3	44.2
**ρ_h (a.u)**	0.56	0.53
**σ_h (nm)**	0.24	0.25
**ρ_c (a.u.)**	0.29	0.34
**σ_c (nm)**	0.75	0.75
**z_h (nm)**	1.84	1.81

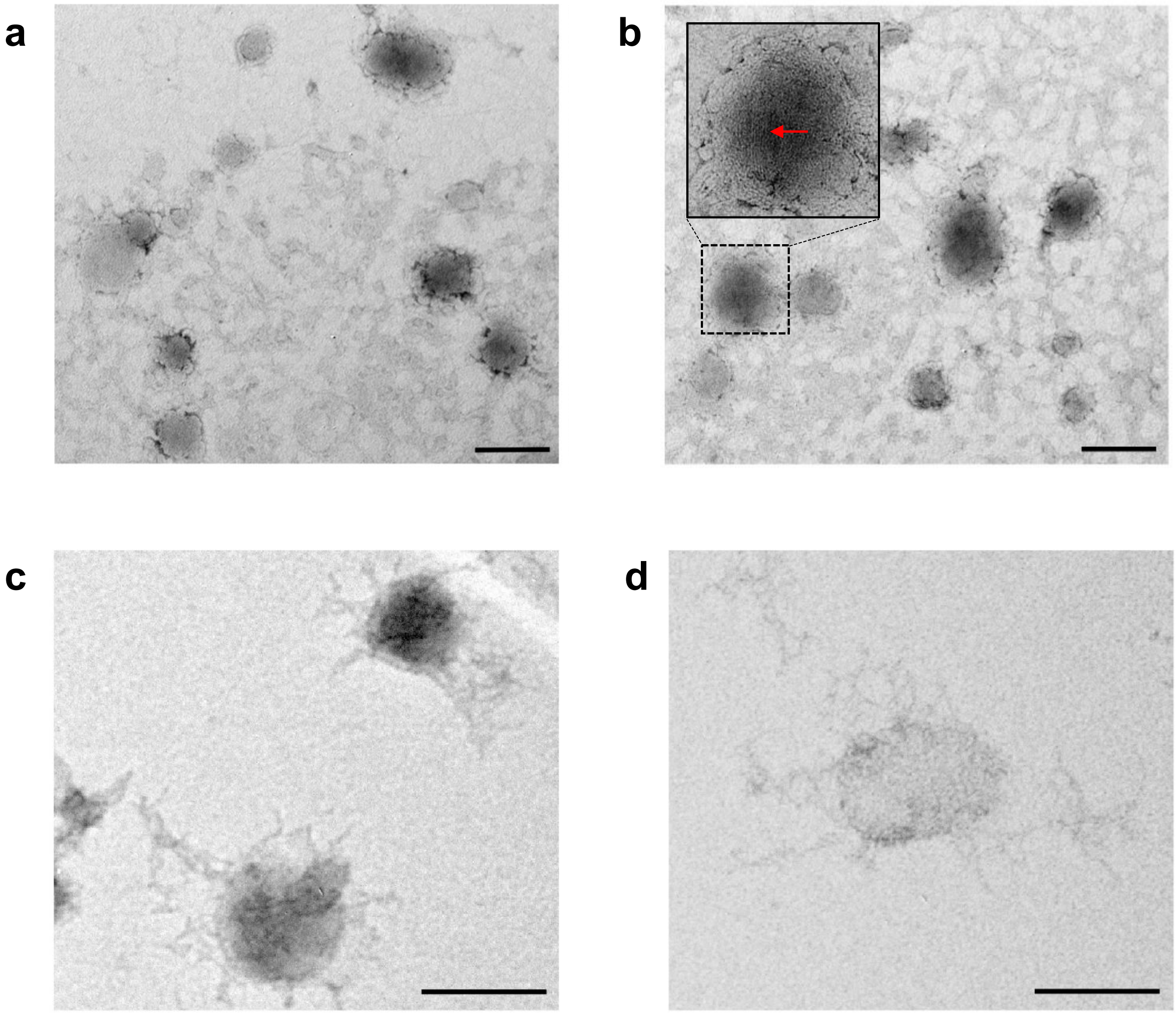

Figure S2. Representative transmission electron microscopy (TEM) images of PLs (panels a and b) and DDLs (panels c and d). In both samples are visible nano-sized, rounded-shaped vesicles. In the inset of panel b, a vesicle is enlarged to make lamellar periodicity visible (indicated by a red arrow). The presence of DNA filaments on the surface is detectable in DDLs. Bars correspond to 200 nm.

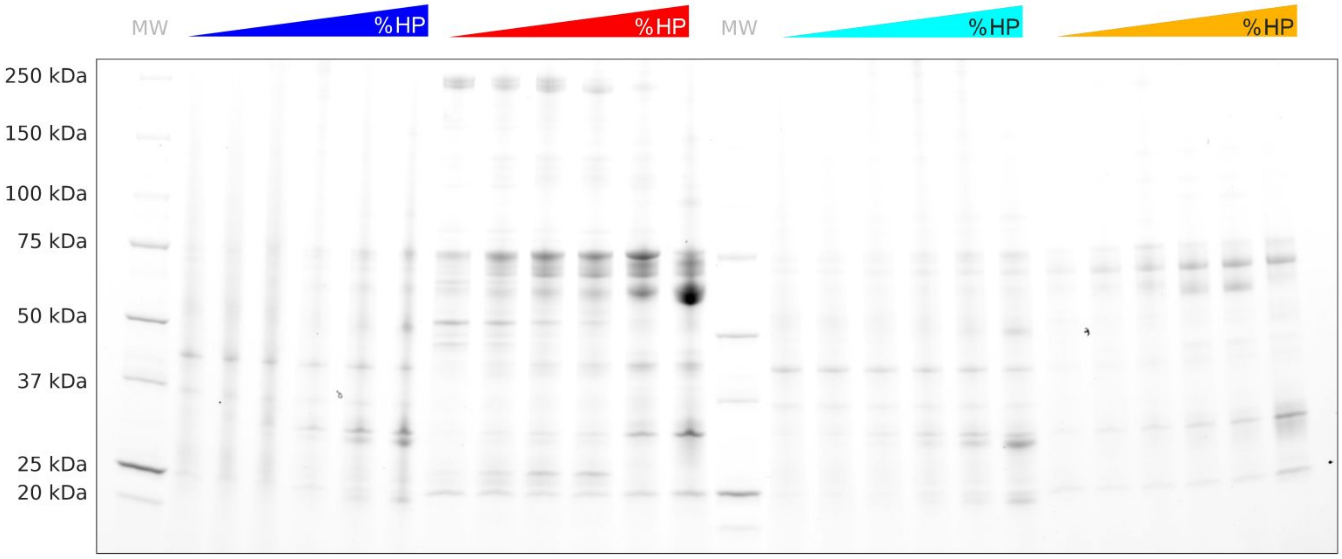

Figure S3. Image for protein corona analysis by 1D SDS-PAGE. (blue) DDL1, (red) PL1, (cyan) DDL2, and (gold) PL2 at increasing amount of human plasma (HP). The corresponding electrophoretic profiles are reported in Fig. 2c.

Table S2. Lists of plasma proteins identified in the coronas of DDLs and PLs by nano-LC-MS/MS.

	DDL1		PL1	DDL2		PL2
Protein	HP=5%	HP=50%	HP=50%	HP=5%	HP=50%	HP=50%
APOC3	4.14%	3.77%	14.70%	3.19%	11.54%	13.61%
APOC2	4.12%	4.54%	20.95%	3.03%	5.22%	6.72%
APOA2	7.77%	2.10%	10.20%	3.01%	1.13%	2.04%
APOA1	7.38%	1.10%	1.93%	7.71%	1.12%	2.35%
APOE	2.25%	14.32%	13.61%	1.47%	4.08%	4.68%
APOC1	10.93%	1.37%	0.28%	14.07%	2.70%	2.39%
ALB	0.62%	3.81%	4.42%	0.75%	4.90%	9.12%
ACTG1	9.81%	4.15%	0.82%	13.27%	4.13%	0.87%
APOD	0.03%	0.23%	3.39%	0.59%	9.17%	15.25%
C1QC	0.22%	9.44%	0.61%	0.09%	7.91%	5.45%
C1QB	0.20%	8.64%	0.46%	0.05%	7.82%	5.70%
HIST1H2BL	5.94%	4.42%	0.42%	6.02%	2.15%	0.49%
IGKC	1.44%	3.19%	1.27%	0.77%	2.77%	4.20%
VTN	1.19%	1.41%	9.15%	0.55%	0.31%	0.72%
IGHM	1.09%	2.47%	0.63%	0.20%	1.36%	1.34%
HMGB1;HMGB1P1	5.63%	1.36%	0.34%	4.59%	0.82%	0.20%
HIST1H1E;HIST1H1D	3.67%	2.38%	0.19%	2.46%	1.04%	0.31%
APOA4	0.85%	0.68%	1.70%	0.99%	0.11%	0.27%
IGHG1	0.60%	2.14%	0.40%	0.23%	1.68%	2.30%
PF4	0.02%	1.26%	1.43%	0.01%	0.88%	3.73%
SERPINA1	0.01%	0.12%	0.13%	0.04%	0.17%	0.36%
IGLL5;IGLC1	0.76%	1.46%	0.51%	0.36%	1.23%	1.50%
KRT1	0.96%	0.56%	0.22%	1.36%	3.31%	0.37%
IGLC3;IGLC2;IGLC6	1.25%	1.46%	0.44%	0.30%	1.29%	1.42%
C4BPA	0.21%	0.22%	1.50%	0.15%	0.72%	2.00%
ACTBL2	1.75%	0.86%	0.10%	1.81%	0.98%	0.06%
C1QA	0.03%	2.59%	0.10%	0.02%	1.67%	1.37%
C3	0.13%	0.52%	1.10%	0.03%	0.20%	0.58%
HIST2H3A	1.43%	0.50%	0.11%	2.07%	0.30%	0.07%
CLU	0.22%	0.33%	0.67%	0.02%	0.35%	0.22%
FGG	0.15%	0.06%	0.06%	0.04%	0.08%	0.18%
IGHG3	0.43%	1.17%	0.13%	0.12%	0.83%	0.67%
FGB	0.05%	0.05%	0.05%	0.02%	0.08%	0.13%
HSPA8	1.04%	0.51%	0.07%	1.24%	0.43%	0.08%
IGHA1	0.10%	0.25%	0.25%	0.01%	0.28%	0.66%
APOB	0.15%	0.11%	0.28%	0.14%	0.08%	0.15%
HMGB2	1.41%	0.47%	0.06%	0.68%	0.16%	0.04%
HNRNPA	0.71%	0.24%	0.05%	1.60%	0.23%	0.03%
FGA	0.13%	0.09%	0.09%	0.07%	0.10%	0.18%
HIST1H2A	0.52%	1.05%	0.06%	0.57%	0.26%	0.08%
C4B	0.06%	0.14%	0.51%	0.01%	0.11%	0.36%
PPIA	0.75%	0.49%	0.06%	0.81%	0.42%	0.10%
KRT10	0.17%	0.15%	0.18%	0.62%	1.18%	0.21%

HIST1H4A	0.78%	0.35%	0.04%	1.14%	0.16%	0.03%
CFP	0.01%	1.26%	0.26%	0.00%	0.80%	0.26%
SAA4	0.37%	0.43%	0.45%	0.32%	0.16%	0.25%
GAPDH	0.74%	0.43%	0.06%	0.75%	0.32%	0.05%
F2	0.01%	0.01%	0.39%	0.00%	0.02%	0.15%
SERPINA3	0.04%	0.22%	0.00%	0.01%	0.31%	0.13%
KRT9	0.12%	0.05%	0.06%	0.17%	1.52%	0.16%
HIST1H1B	0.70%	0.39%	0.02%	0.48%	0.20%	0.04%
ITIH2	0.04%	0.01%	0.08%	0.01%	0.01%	0.06%
HSP90AA1	0.53%	0.30%	0.04%	0.68%	0.30%	0.04%
ENO1	0.54%	0.26%	0.05%	0.73%	0.22%	0.04%
PKM	0.38%	0.34%	0.07%	0.70%	0.31%	0.05%
GSN	0.70%	0.04%	0.02%	0.12%	0.06%	0.02%
MSN	0.43%	0.17%	0.04%	0.80%	0.16%	0.02%
HP	0.02%	0.21%	0.12%	0.05%	0.17%	0.41%
SAA1	0.04%	0.05%	0.48%	0.23%	0.24%	0.37%
PLG	0.26%	0.28%	0.05%	0.33%	0.38%	0.05%
CRP	0.00%	0.40%	0.58%	0.00%	0.16%	0.45%
HNRNPA3	0.32%	0.15%	0.02%	0.87%	0.12%	0.01%
CORO1A	0.31%	0.18%	0.04%	0.76%	0.20%	0.02%
TTR	0.00%	0.02%	0.04%	0.00%	0.01%	0.05%
PON1	0.01%	0.00%	0.17%	0.00%	0.00%	0.02%
HNRNPA2B1	0.27%	0.13%	0.04%	0.69%	0.13%	0.02%
EEF1A1P5	0.37%	0.14%	0.03%	0.55%	0.14%	0.03%
PTMA	0.31%	0.09%	0.07%	0.39%	0.01%	0.00%
CFH	0.02%	0.59%	0.02%	0.00%	0.19%	0.12%
APOH	0.01%	0.06%	0.01%	0.02%	0.63%	0.43%
PROS1	0.01%	0.02%	0.16%	0.00%	0.06%	0.38%
AMBP	0.00%	0.01%	0.04%	0.00%	0.00%	0.02%
NME2;NME2P1	0.42%	0.09%	0.01%	0.46%	0.08%	0.01%
PGK1	0.26%	0.19%	0.03%	0.36%	0.18%	0.03%
RPS23	0.27%	0.07%	0.02%	0.55%	0.07%	0.00%
CP	0.00%	0.01%	0.03%	0.00%	0.01%	0.03%
IGHG2	0.09%	0.19%	0.07%	0.01%	0.14%	0.36%
IGKV3-20	0.10%	0.24%	0.03%	0.01%	0.21%	0.15%
TF	0.03%	0.12%	0.09%	0.03%	0.18%	0.37%
HSP90AB1	0.28%	0.14%	0.02%	0.33%	0.12%	0.02%
APOM	0.00%	0.04%	0.51%	0.02%	0.00%	0.07%
EEF2	0.22%	0.09%	0.02%	0.39%	0.08%	0.01%
PA2G4	0.25%	0.09%	0.01%	0.34%	0.09%	0.01%
CFL1	0.15%	0.22%	0.02%	0.21%	0.16%	0.03%
JCHAIN	0.06%	0.14%	0.02%	0.03%	0.08%	0.05%
PSMA6	0.21%	0.10%	0.02%	0.27%	0.09%	0.01%
ALDOA	0.24%	0.07%	0.01%	0.29%	0.08%	0.01%
AHSG	0.00%	0.00%	0.01%	0.00%	0.01%	0.01%
RPS3A	0.20%	0.09%	0.01%	0.35%	0.06%	0.01%
APOC4	0.10%	0.13%	0.08%	0.06%	0.13%	0.05%
FN1	0.02%	0.01%	0.01%	0.00%	0.02%	0.02%

CFB	0.06%	0.00%	0.00%	0.00%	0.01%	0.01%
KRT14	0.01%	0.00%	0.02%	0.06%	0.59%	0.01%
APCS	0.08%	0.02%	0.01%	0.01%	0.02%	0.01%
PRDX1	0.21%	0.12%	0.01%	0.21%	0.12%	0.02%
IGKV3	0.01%	0.09%	0.03%	0.04%	0.08%	0.09%
KNG1	0.01%	0.01%	0.03%	0.00%	0.01%	0.02%
ORM2	0.00%	0.01%	0.01%	0.00%	0.01%	0.02%
KRT2	0.03%	0.04%	0.05%	0.17%	0.27%	0.07%
A2M	0.01%	0.04%	0.02%	0.02%	0.02%	0.05%
PCNA	0.18%	0.07%	0.01%	0.23%	0.06%	0.00%
CAPZB	0.15%	0.07%	0.01%	0.24%	0.07%	0.01%
CD5L	0.02%	0.13%	0.04%	0.00%	0.08%	0.07%
YWHAZ	0.19%	0.06%	0.00%	0.21%	0.07%	0.01%
ITIH1	0.00%	0.00%	0.02%	0.00%	0.00%	0.01%
HNRNPU	0.16%	0.06%	0.01%	0.22%	0.05%	0.00%
EEF1G	0.15%	0.05%	0.01%	0.24%	0.05%	0.00%
ANP32B	0.19%	0.05%	0.02%	0.16%	0.06%	0.00%
APOL1	0.22%	0.03%	0.02%	0.06%	0.02%	0.01%
RHOA	0.14%	0.02%	0.00%	0.26%	0.02%	0.00%
HNRNPD	0.06%	0.06%	0.01%	0.25%	0.05%	0.01%
HPR	0.02%	0.01%	0.00%	0.00%	0.02%	0.00%
RNASE4	0.00%	0.03%	0.10%	0.00%	0.04%	0.29%
HBB;HBD	0.11%	0.16%	0.10%	0.01%	0.05%	0.02%
C4BPB	0.01%	0.00%	0.11%	0.03%	0.03%	0.18%
NCL	0.23%	0.06%	0.02%	0.10%	0.03%	0.01%
CAPZA2	0.12%	0.10%	0.01%	0.11%	0.10%	0.01%
SIGLEC16	0.00%	0.00%	0.00%	0.00%	0.00%	0.00%
ANP32A	0.16%	0.07%	0.01%	0.09%	0.04%	0.01%
IGHV3	0.04%	0.13%	0.03%	0.01%	0.11%	0.11%
FSCN1	0.14%	0.05%	0.00%	0.18%	0.04%	0.00%
RBBP4	0.14%	0.06%	0.01%	0.13%	0.06%	0.00%
PRH1	0.00%	0.00%	0.00%	0.13%	0.29%	0.00%
LDHB	0.11%	0.05%	0.01%	0.16%	0.05%	0.01%
WDR1	0.12%	0.07%	0.00%	0.15%	0.05%	0.01%
ARPC2	0.12%	0.03%	0.00%	0.21%	0.03%	0.00%
RPL11	0.13%	0.06%	0.01%	0.14%	0.04%	0.01%
MDH1	0.11%	0.05%	0.01%	0.17%	0.04%	0.01%
ORM1	0.00%	0.02%	0.01%	0.00%	0.01%	0.02%
SNRPD1	0.13%	0.08%	0.00%	0.09%	0.07%	0.00%
RPL22	0.19%	0.00%	0.00%	0.17%	0.00%	0.00%
SERPING1	0.00%	0.00%	0.00%	0.00%	0.00%	0.01%
ACTA1	0.10%	0.05%	0.01%	0.12%	0.07%	0.02%
SPP2	0.00%	0.00%	0.28%	0.00%	0.00%	0.00%
RPL12	0.04%	0.02%	0.00%	0.04%	0.01%	0.00%
LCAT	0.00%	0.00%	0.06%	0.00%	0.06%	0.18%
PDIA3	0.11%	0.02%	0.00%	0.16%	0.03%	0.00%
TPI1	0.11%	0.03%	0.00%	0.14%	0.03%	0.00%
H1FX	0.12%	0.08%	0.00%	0.07%	0.03%	0.01%

ARPC1B	0.10%	0.03%	0.01%	0.16%	0.02%	0.00%
TPM3;TPM1;TPM2	0.09%	0.03%	0.00%	0.12%	0.01%	0.00%
ATIC	0.08%	0.03%	0.01%	0.15%	0.03%	0.00%
CFHR1	0.00%	0.13%	0.00%	0.00%	0.06%	0.13%
RAN	0.06%	0.03%	0.00%	0.17%	0.04%	0.00%
HSPA1B;HSPA1A	0.09%	0.03%	0.01%	0.14%	0.03%	0.00%
IGKV2	0.01%	0.02%	0.02%	0.02%	0.03%	0.09%
IGFALS	0.13%	0.01%	0.00%	0.02%	0.01%	0.01%
SH3BGRL3	0.07%	0.10%	0.00%	0.02%	0.08%	0.03%
AHCY	0.09%	0.03%	0.00%	0.14%	0.02%	0.00%
ARHGDIA	0.09%	0.03%	0.00%	0.14%	0.03%	0.00%
ITIH4	0.01%	0.01%	0.00%	0.01%	0.01%	0.01%
EZR	0.05%	0.05%	0.00%	0.13%	0.03%	0.00%
PRDX4	0.08%	0.04%	0.00%	0.07%	0.04%	0.01%
HBA1	0.03%	0.08%	0.03%	0.03%	0.03%	0.03%
IGHV3-7	0.00%	0.10%	0.04%	0.00%	0.01%	0.04%
FABP5	0.07%	0.02%	0.00%	0.13%	0.04%	0.00%
RCC2	0.06%	0.02%	0.00%	0.13%	0.02%	0.00%
DEK	0.08%	0.02%	0.00%	0.09%	0.01%	0.00%
PSMB1	0.08%	0.04%	0.01%	0.07%	0.03%	0.00%
RPL5	0.06%	0.04%	0.00%	0.10%	0.02%	0.00%
PSMA2	0.08%	0.03%	0.01%	0.09%	0.02%	0.00%
SET;SETSIP	0.12%	0.01%	0.00%	0.06%	0.01%	0.00%
KRT16	0.01%	0.00%	0.01%	0.01%	0.20%	0.00%
KRT5	0.01%	0.00%	0.00%	0.04%	0.17%	0.01%
C1S	0.01%	0.01%	0.01%	0.00%	0.01%	0.02%
SNRPG;SNRPGP15	0.10%	0.02%	0.00%	0.08%	0.01%	0.00%
COTL1	0.10%	0.01%	0.00%	0.09%	0.01%	0.00%
ACTR3	0.03%	0.03%	0.00%	0.11%	0.02%	0.00%
PSMA5	0.07%	0.03%	0.00%	0.08%	0.02%	0.00%
ARHGDIB	0.05%	0.02%	0.01%	0.11%	0.02%	0.00%
NPM1	0.09%	0.03%	0.01%	0.06%	0.01%	0.01%
YWHAB;YWHAQ	0.06%	0.02%	0.00%	0.09%	0.02%	0.00%
SERPIND1	0.00%	0.00%	0.00%	0.00%	0.00%	0.00%
LYZ	0.01%	0.08%	0.01%	0.01%	0.07%	0.02%

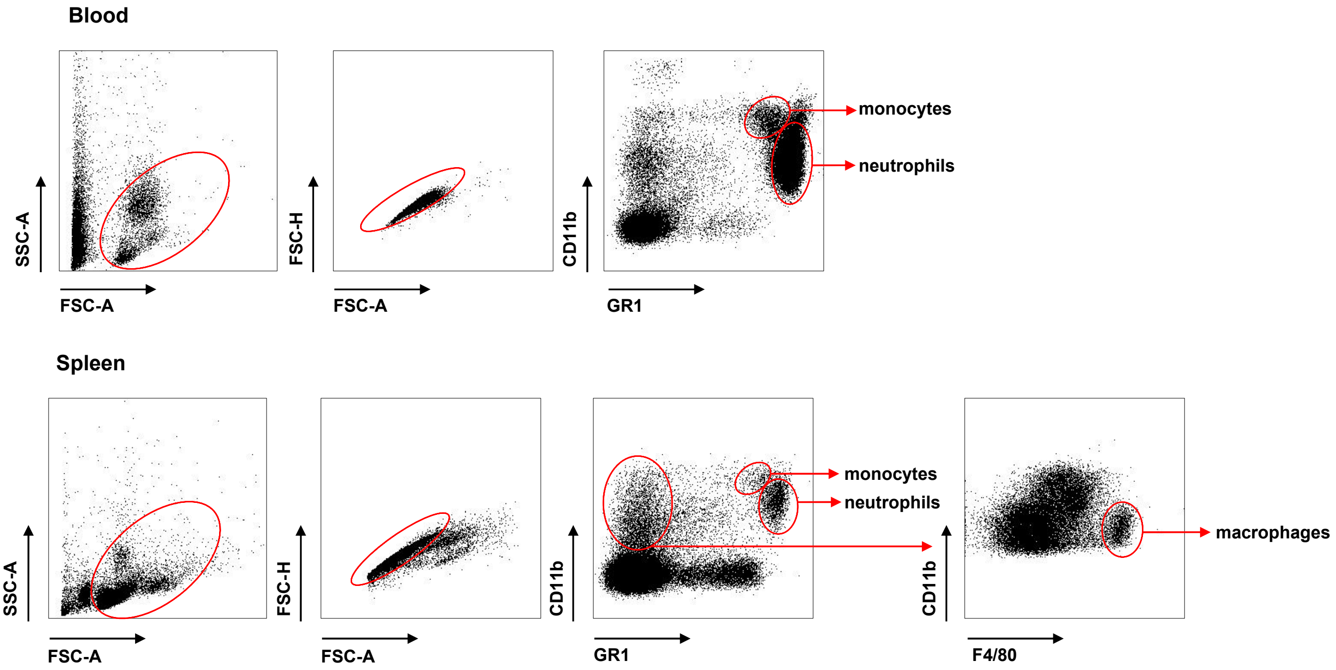

Figure S4. Gating strategy of mouse phagocyte populations. Representative dot plots of phagocyte populations in the blood and in the spleen dissected by gating on CD11b^{+}GR1^{low} (monocytes), CD11b GR1^{high} (neutrophils) and CD11b^{+}GR1^{-}F4/80^{+} (macrophages).

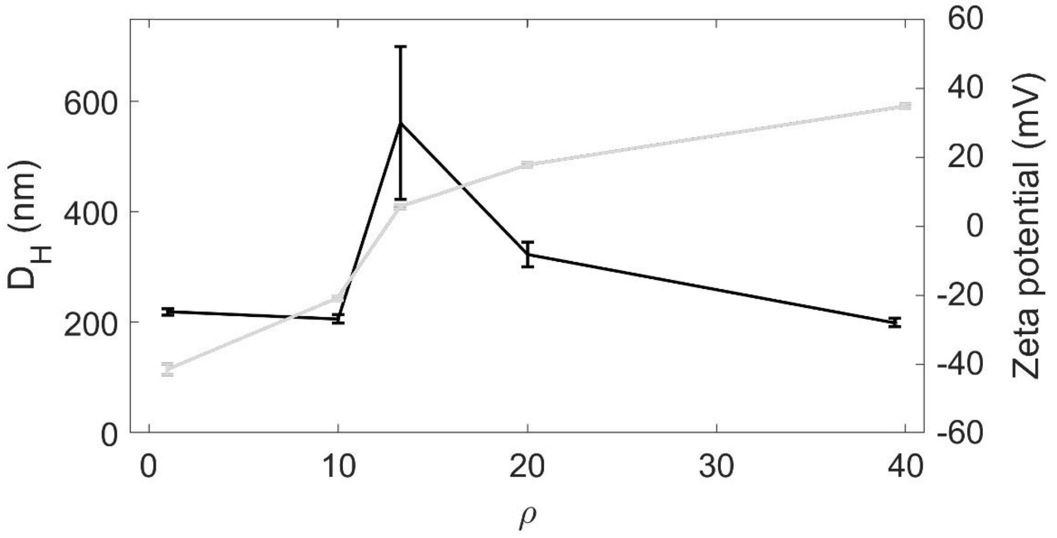

Figure S5. Characterization of CL2/oligonucleotide (ON) lipoplexes as a function of the cationic lipid/ON weight ratio, ρ. Size (black points) and zeta potential (grey points) of lipoplexes prepared by mixing CL2 and ON as a function of ρ. Comparing results of Fig. S5 with those reported in Fig. 2, we observe that both the curves are shifted to higher ρ values. This result is in full agreement with the conclusions of previous investigations showing that more cationic lipid is needed to complex ONs with respect to that needed to condense plasmid DNA (Munoz-Úbeda, Mónica, et al. "Why is less cationic lipid required to prepare lipoplexes from plasmid DNA than linear DNA in gene therapy?." Journal of the American Chemical Society 133.45 (2011): 18014-18017 [1]). Moreover, at fixed DNA mass, ONs bear double the nucleotides than plasmid DNA. Thus, to use the same moles of nucleotides in all the animal experiments, we employed double the mass of cationic lipid when the ON is used (i.e., $\rho = 2$). At $\rho = 2$ DDL2 were small in size ($D_H \approx 200$ nm), and negatively charged (zeta potential ≈ - 40 mV). ON-decorated lipoplexes from CL2 were indicated as DDL2* to differentiate them from those decorated with plasmid DNA) (i.e., DDL2).

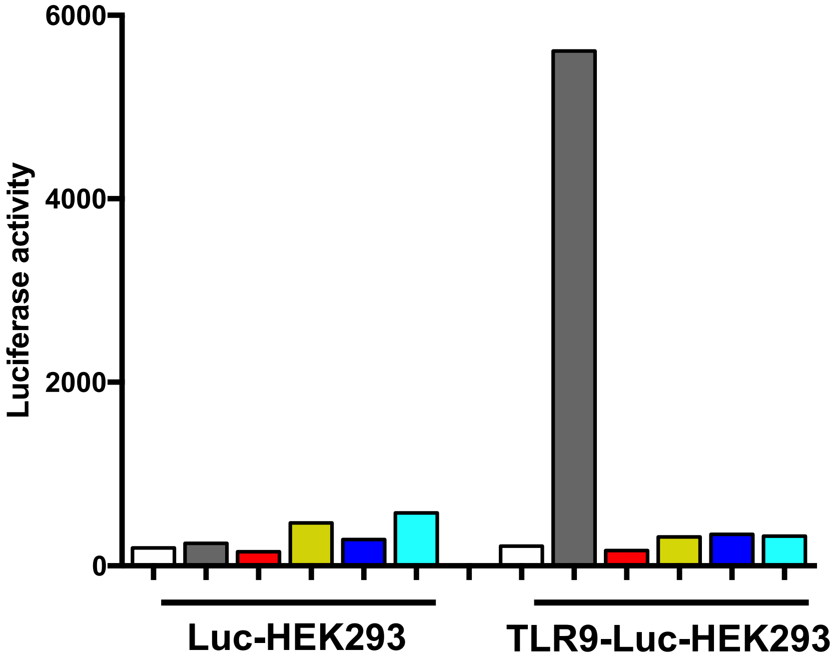

Figure S6. Lipoplexes do not trigger TLR9 activity in reporter cells. HEK293/Luc reporter cells transfected or not with TLR9 were incubated for 5 hours with PL1, PL2, DDL1, DDL2 or specific TLR9 agonist. Data are expressed as luciferase activity. A representative experiment out of two is shown. Color code: untreated cells (white), TLR9 agonist (dark grey), DDL1 (blue), DDL2 (cyan), PL1 (red), PL2 (gold).

**Supplementary Table S3. Material characterization Question**	**Yes**	**No**
1.1 Are "**best reporting practices**" **available** for the nanomaterial used?	Not applicable	
1.2 If they are available, **are they used**? If not available, ignore this question and proceed to the next one.		
1.3 Are extensive and clear instructions reported detailing all steps of **synthesis** and the resulting **composition** of the nanomaterial?	√	
1.4 Is the **size** (or **dimensions**, if non-spherical) and **shape of** the nanomaterial reported?	√	
1.5 Is the **size dispersity** or **aggregation** of the nanomaterial reported?	√	
1.6 Is the **zeta potential** of the nanomaterial reported?	√	
1.7 Is the **concentration (mass/volume)** of the nanomaterial reported?	√	
1.8 Is the amount of any **drug loaded** reported? 'Drug' here broadly refers to functional cargos (*e.g.*, proteins, small molecules, nucleic acids).	√	
1.9 Is the **targeting performance** of the nanomaterial reported, including **amount** of ligand bound to the nanomaterial if the material has been functionalised through addition of targeting ligands?	Not applicable	
1.10 Is the **label signal** per nanomaterial/particle reported? For example, fluorescence signal per particle for fluorescently labeled nanomaterials.	√	
1.11 If a material property not listed here is varied, has it been **quantified**?	Not applicable	
1.12 Were characterizations performed in a **fluid mimicking biological conditions**?	√	
1.13 Are details of how these parameters were **measured/estimated** provided?	√	

**Supplementary Table 3. Biological characterization Question**	**Yes**	**No**
2.1 Are **cell seeding details**, including **number of cells plated, confluency at start of experiment,** and **time between seeding and experiment** reported?	√	
2.2 If a standardised cell line is used, are the **designation and source** provided?	√	
2.3 Is the **passage number** (total number of times a cell culture has been subcultured) known and reported?	Not applicable	
2.4 Is the last instance of **verification of cell line** reported? If no verification has been performed, is the time passed and passage number since acquisition from trusted source (*e.g.*, ATCC or ECACC) reported? For information, see *Science* **347** (2015) 938; http://doi.org/10.1126/science.347.6225.938	No	
2.5 Are the results from **mycoplasma testing** of cell cultures reported?	No	
2.6 Is the **background signal of cells/tissue** reported? (*E.g.*, the fluorescence signal of cells without particles in the case of a flow cytometry experiment.)	√	
2.7 Are **toxicity studies** provided to demonstrate that the material has the expected toxicity, and that the experimental protocol followed does not?	Not applicable	
2.8 Are details of media preparation (**type of media, serum,** any **added antibiotics**) provided?	√	
2.9 Is a **justification of the biological model** used provided? For examples for cancer models, see *Cancer Res.* **75** (2015) 4016; http://doi.org/10.1158/0008-5472.CAN-15-1558, and *Mol. Ther.* **20** (2012) 882; http://doi.org/10.1038/mt.2012.73, and *ACS Nano* **11** (2017) 9594; http://doi.org/10.1021/acsnano.7b04855	√	
2.10 Is characterization of the **biological fluid** (*ex vivo*/*in vitro*) reported? For example, when investigating protein adsorption onto nanoparticles dispersed in blood serum, pertinent aspects of the blood serum should be characterised (*e.g.*, protein concentrations and differences between donors used in study).	√	
2.11 For **animal experiments**, are the ARRIVE guidelines followed? For details, see *PLOS Biol.* **8** (2010) e1000412; http://doi.org/10.1371/journal.pbio.1000412	√	

**Supplementary Table 3. Experimental details Question**	**Yes**	**No**
3.1 For cell culture experiments: are **cell culture dimensions** including **type of well**, **volume of added media**, reported? Are cell types (*i.e.*; adherent *versus* suspension) and **orientation** (if non-standard) reported?	√	
3.2 Is the **dose of material administered** reported? This is typically provided in nanomaterial mass, volume, number, or surface area added. Is sufficient information reported so that regardless of which one is provided, the other dosage metrics can be calculated (*i.e.* using the dimensions and density of the nanomaterial)?	√	
3.3 For each type of imaging performed, are details of how **imaging** was performed provided, including details of **shielding, non-uniform image processing**, and any **contrast agents** added?	Not applicable	
3.4 Are details of how the dose was administered provided, including **method of administration, injection location, rate of administration,** and details of **multiple injections**?	√	
3.5 Is the methodology used to **equalise dosage** provided?	√	
3.6 Is the **delivered dose** to tissues and/or organs (*in vivo*) reported, as % injected dose per gram of tissue (%ID g^{-1})?	√	
3.7 Is **mass of each organ/tissue measured** and **mass of material** reported?	Not applicable	
3.8 Are the **signals of cells/tissues with nanomaterials** reported? For instance, for fluorescently labeled nanoparticles, the total number of particles per cell or the fluorescence intensity of particles + cells, at each assessed timepoint.	Not applicable	
3.9 Are **data analysis details**, including **code used** for analysis provided?	√	
3.10 Is the **raw data** or **distribution of values** underlying the reported results provided? For examples, see *R. Soc. Open Sci.* **3** (2016) 150547; http://doi.org/10.1098/rsos.150547, https://opennessinitiative.org/making-your-data-public/, http://journals.plos.org/plosone/s/data-availability, and https://www.nature.com/sdata/policies/repositories	√	

1. Munoz-Úbeda, M.; Misra, S. K.; Barrán-Berdón, A. L.; Aicart-Ramos, C.; Sierra, M. B.; Biswas, J.; Kondaiah, P.; Junquera, E.; Bhattacharya, S.; Aicart, E., Why is less cationic lipid required to prepare lipoplexes from plasmid DNA than linear DNA in gene therapy? *Journal of the American Chemical Society* **2011,** *133* (45), 18014-18017.
